# Venous excess and lung ultrasound during continuous renal replacement therapy in critically ill patients (VExLUS-RRT): a prospective observational study

**DOI:** 10.1016/j.aicoj.2026.100129

**Published:** 2026-07-30

**Authors:** Thanphisit Trakarnvanich, Anyarin Wannakittirat, Sadudee Peerapornratana, Yingyos Avihingsanon, Marlies Ostermann, Nattachai Srisawat, Nuttha Lumlertgul

**Affiliations:** a Division of Nephrology, Department of Medicine, Faculty of Medicine, Chulalongkorn University, Bangkok, Thailand; b Excellence Centre for Critical Care Nephrology, King Chulalongkorn Memorial Hospital, Thai Red Cross Society, Bangkok, Thailand; c Centre of Excellence in Critical Care Nephrology, Faculty of Medicine, Chulalongkorn University, Bangkok, Thailand; d Division of Nephrology, Department of Medicine, Faculty of Medicine Vajira Hospital, Navamindradhiraj University, Bangkok, Thailand; e Division of Nephrology, Department of Medicine, Faculty of Medicine, Naresuan University Hospital, Phitsanulok, Thailand; f Department of Laboratory Medicine, Faculty of Medicine, Chulalongkorn University, Bangkok, Thailand; g Department of Critical Care, Guy’s and St Thomas’ National Health Service Foundation Trust, King’s College, London, United Kingdom; h Academy of Science, Royal Society of Thailand, Bangkok, Thailand

**Keywords:** Acute kidney injury, Lung ultrasound, Point-Of-Care ultrasound, Venous excess ultrasound, Continuous renal replacement therapy

## Abstract

**Background:**

No gold standard currently exists to evaluate fluid status during continuous renal replacement therapy (CRRT). We aimed to describe systemic venous congestion by using venous excess ultrasound (VExUS) and pulmonary congestion by lung ultrasound (LUS) in critically ill patients with acute kidney injury (AKI) who received CRRT.

**Methods:**

This was a single-centre prospective cohort study. Adult patients with AKI undergoing CRRT underwent serial ultrasound assessments on Day 1 and 3 by two independent operators. VExUS score and its individual components, including an inferior vena cava (IVC) diameter (≥2 cm), hepatic vein, portal vein, as well as intrarenal vein Doppler flow patterns, and 6-zone lung ultrasound (LUS) scores (ranged from 0 to 20 points), were collected. An exploratory modified VExUS (mVExUS) grading was derived by incorporating an IVC distensibility index (<18%) or collapsibility index (<50%). Venous congestion was defined as VExUS or mVExUS Grade ≥2. The primary descriptive outcome was the prevalence of venous congestion and serial LUS score changes. The principal clinical endpoint was the association between ultrasound parameters and 90-day mortality **(NCT06254703).**

**Results:**

Among 100 enrolled patients (median age 66 years, male 58%), baseline venous congestion was documented in 20% by VExUS score and 35% by mVExUS scores. The median LUS score was 4 (interquartile range 1–9). Baseline VExUS Grade 2−3 (Log rank p = 0.049) and mVExUS Grade 2−3 (Log rank p = 0.01) were associated with lower 90-day survival. In time-updated Cox models after adjustment for baseline Sequential Organ Failure Assessment score, hepatic vein Doppler Grade 2−3 (adjusted hazard ratio (aHR) 1.78; 95% confidence interval (CI) 1.05–3.01, p = 0.01) and higher LUS scores (aHR 1.04; 95% CI 1.001–1.08, p = 0.04) were independently associated with 90-day mortality. Unchanged or worsening LUS scores between Day 1 and Day 3 were associated with a significantly higher cumulative fluid balance compared to improved scores (p = 0.01).

**Conclusion:**

Systemic and pulmonary congestion assessed by multiorgan point-of-care ultrasound was associated with increased 90-day mortality in AKI patients receiving CRRT. Future studies should explore the utility of these tools in guiding individualised fluid management during CRRT.

## Highlights

• Venous congestion occurred in 20% (VExUS) and 35% (modified VExUS) of AKI patients at CRRT initiation.
• Grade 2–3 VExUS and modified VExUS at Day 1 were associated with increased risks of 90-day mortality (p = 0.049, p = 0.01).
• Dynamic changes in cumulative fluid balance correlated with changes in LUS scores between Day 1 and 3.

## Graphical abstract

**Figure:**
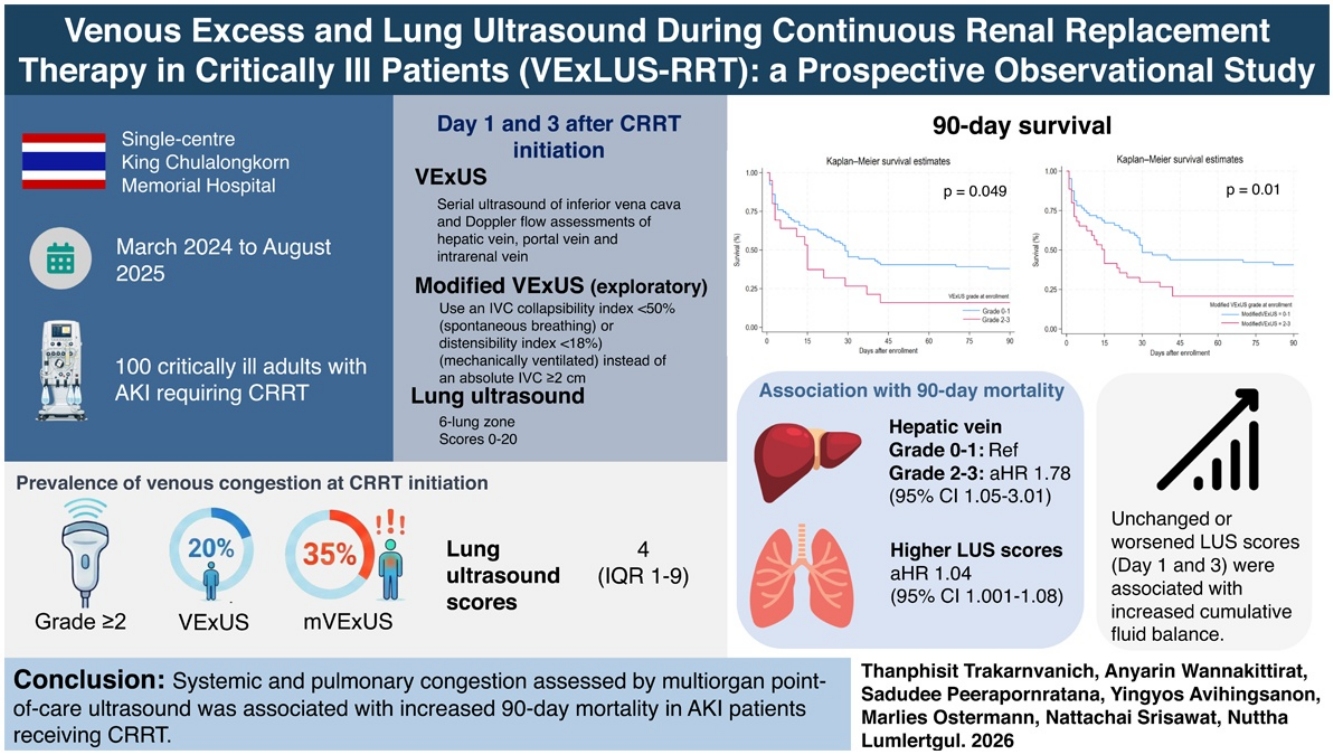

## Introduction

Both hypovolaemia and fluid accumulation syndrome (FAS) are associated with higher morbidity and mortality in critically ill patients [1]. FAS is defined as any degree of fluid accumulation with new-onset organ dysfunction, including pulmonary, cardiovascular, gastrointestinal, and renal systems [2]. Renal venous congestion is a well-recognised driver of acute kidney injury (AKI) due to impaired venous outflow [3]. Nevertheless, a gold standard for monitoring and diagnosing venous congestion during AKI, especially during continuous renal replacement therapy (CRRT), has yet to be established. Clinical signs such as peripheral oedema, hepatomegaly and pulmonary crackles have limited sensitivity and specificity [4,5]. Objective parameters such as central venous pressure (CVP), changes in body weight and cumulative fluid balance (CFB) are frequently used but can be confounded by several factors including underlying disease processes and dynamic changes inherent to critical care.

More advanced tools, such as point-of-care ultrasound (POCUS), bioimpedance analysis (BIA) and cardiac output monitoring, are sometimes employed to provide further insights during the de-resuscitation phase [2,6]. Venous Excess Ultrasound (VExUS) is a grading system developed to evaluate systemic venous congestion [7]. VExUS has been shown to predict the development of AKI in post-cardiac surgery patients [8]. Previous studies have reported an association between VExUS score and renal replacement therapy (RRT)-free days in AKI [9,10]. In critically ill patients with cardiorenal syndrome, VExUS-guided fluid management improved the likelihood of decongestion [11]. Following fluid removal by intermittent hemodialysis, the degree of venous congestion improved in end-stage kidney disease (ESKD) patients with elevated VExUS scores [12]. In addition, lung ultrasound (LUS) can help detect pulmonary congestion and has been shown to correlate with hypoxemia in AKI patients [13].

Few studies have described the utility of VExUS and LUS in critically ill patients with severe AKI, particularly in those treated with CRRT [10]. Therefore, we aimed to characterise venous and pulmonary congestion by VExUS grading and LUS scores in this population. Recognising that an IVC diameter may be confounded by factors such as right ventricular function, intrathoracic pressure and intraabdominal pressure, we also evaluated an exploratory modified VExUS (mVExUS) score by utilising an IVC distensibility index <18% (for mechanically ventilated patients) or the IVC collapsibility index <50% (for spontaneously breathing patients), instead of an absolute IVC diameter threshold [14,15]. The primary descriptive outcome was the presence of venous congestion (using VExUS or mVExUS) and pulmonary congestion (by LUS) at Day 1 and Day 3 during CRRT. Our principal clinical endpoint of interest was the association between ultrasound parameters and 90-day mortality.

## Methods

### Study design and setting

This was a single-centre prospective observational study at King Chulalongkorn Memorial Hospital between March 1, 2024 and August 30, 2025. The study was approved by the Ethics Committee at the Faculty of Medicine, Chulalongkorn University (0899/66) and registered on ClinicalTrials.gov (**NCT06254703).** The study adhered to the Declaration of Helsinki of 2024 and is reported according to the STrengthening the Reporting of OBservational Studies in Epidemiology (STROBE) guideline [16]. All participants or legal representatives provided written informed consent.

### Participants

We included critically ill patients aged ≥18 years who had AKI by Kidney Disease: Improving Global Outcomes (KDIGO) Criteria [17] and who received CRRT as determined by the primary clinical team based on conventional indications, including severe hyperkalaemia, metabolic acidosis, pulmonary oedema, uraemic symptoms, blood urea nitrogen level ≥112 mg/dL, or oliguria ≥72 h. Patients were excluded from the study if they (1) had ESKD, (2) were kidney transplant recipients, (3) received RRT before ICU admission, (4) had structural kidney diseases that would interfere with intrarenal Doppler ultrasound, e.g. renal artery stenosis, autosomal dominant polycystic kidney disease, (5) had conditions that may interfere with portal Doppler assessment, including liver cirrhosis or severe tricuspid regurgitation with structural heart disease, (6) had an underlying disease process with a life expectancy less than 90 days, (7) were pregnant, (8) had acute severe respiratory distress syndrome (ARDS), (9) had an expected life expectancy <48 h, or (10) received extracorporeal membrane oxygenation (ECMO).

Measurements (Detailed in Additional File: Additional methods)

#### Ultrasound measurements

Ultrasonography was performed using a CX-50 (Philips Healthcare, Amsterdam, The Netherlands) or Venue Go™ (GE Healthcare) ultrasound machine, with a phased array or convex array transducer. All ultrasound measurements were collected within 4 h of CRRT initiation (Day 1) and repeated at 72 h (Day 3) after enrollment.

#### VExUS grading

Assessment of venous congestion was performed using Doppler-based techniques as previously described [7]. Patients were positioned in a semi-recumbent dorsal decubitus position (head of the bed at 0–30 °). Concurrent electrocardiogram was used to identify the phases of the cardiac cycle. All measurements were performed at the end of expiration. The hepatic venous, portal venous, and intrarenal venous Doppler flow patterns were obtained and graded on a scale of 1−3. The overall VExUS grade was then categorised based on the Doppler appearances and the IVC diameter into VExUS Grade 0−1 (normal/mild congestion) and Grade 2−3 (moderate/severe congestion). Additionally, portal vein pulsatility fraction (PPF) was calculated as [(peak velocity-minimum velocity) × 100%/peak velocity].

#### Modified VExUS (mVExUS) grading

The conventional VExUS grading incorporates inferior vena cava (IVC) diameter using a fixed cutoff of ≥2.0 cm to define dilatation [7]. Recognising that IVC size may be smaller in Asian populations and can be confounded by various factors [18], we evaluated an exploratory mVExUS criteria which incorporated dynamic indices of IVC rather than an absolute diameter threshold [14,15]. Accordingly, IVC congestion in mVExUS was defined by either an IVC collapsibility index <50% in spontaneously breathing patients or an IVC distensibility index <18% in mechanically ventilated patients, as previously described in the literature [14,15].

#### Lung ultrasound score

Lung ultrasound was performed using a curvilinear ultrasound probe or a phased array probe at 6 different areas [19]. LUS score ranged from 0−20 points.

#### Bioelectrical impedance analysis (BIA)

BIA was performed concurrently with ultrasound assessments on Day 1 using a Body Composition Monitor (Fresenius Medical Care), which employs a multi-frequency Bioelectrical Impedance Spectroscopy technique to measure the body's resistance to a weak alternating electrical current. Body weight was measured using beds with integrated scales (Electric Bed 5 Function Weighting Scale WRA I D). Overhydration (L) was reported.

### Blinding

To ensure the observational nature of the study, the clinical team was blinded to the results of the measurements. These results were not used to guide or alter fluid management decisions during CRRT.

### Reproducibility

Two investigators trained in critical care ultrasound (TT, AW or NL) performed the ultrasound assessments simultaneously and independently. Inter-rater agreement was reported as the percentage of agreement for dichotomous outcomes (no-mild congestion/moderate-severe congestion) using weighted kappa. To account for potential prevalence effects, Prevalence-Adjusted Bias-Adjusted Kappa (PABAK) and Gwet’s Agreement Coefficient (AC) were additionally calculated. A third investigator reviewed the ultrasound images and resolved any inconsistencies in interpretation. The feasibility of obtaining each VExUS component was also recorded.

### Data collection

Baseline characteristics, including demographic information, comorbidities, admission diagnosis and severity of illness by Sequential Organ Failure Assessment (SOFA) score and Acute Physiology And Chronic Health Evaluation II (APACHE II) score were collected. Clinical information was collected including haemodynamic parameters, use of mechanical ventilation, use of vasopressors, central venous pressure (CVP), indications for CRRT initiation, CFB (% relative to body weight on admission) and total ultrafiltration (mL). Total ultrafiltration (mL) per day was defined as the total volume of fluid removed from the patient in the previous 24 h (0600am-0600am). Net ultrafiltration rate (mL/hour) was defined as the volume of fluid removed from the patient minus the volume of fluid infused to the patient per unit of time [20]. N-terminal pro-B-type natriuretic peptide (NT-proBNP) using electrochemiluminescence immunoassay and urine neutrophil gelatinase-associated lipocalin (NGAL) using enzyme-linked immunosorbent assay were reported, if available. Echocardiography was performed by cardiologists. Obtained parameters included tricuspid annular plane systolic excursion (TAPSE), tricuspid annular systolic velocity (S’), left ventricular ejection fraction (LEVF), left ventricular outflow tract velocity-time integral (VTI), left ventricular fractional shortening (%), mitral early to late diastolic filling (MV E:A ratio), E/septal and lateral E’ ratio and right ventricular systolic pressure (RVSP). Follow-up information included CFB (%) and total ultrafiltration (mL) on Day 1 and Day 3, ICU-free days, RRT-free days, vasopressor-free days, ventilator-free days and dialysis dependence status at 28 and 90 days. Survival was followed up until day 90.

### Outcomes

The primary descriptive outcome was the presence of venous congestion, defined as VExUS or mVExUS scores ≥2, and pulmonary congestion by LUS scores at enrolment (Day 1) or on Day 3. The principal clinical endpoint of interest was 90-day mortality. Secondary outcomes included: (1) the association between VExUS, mVExUS, LUS scores and clinical outcomes including 28-day and 90-day mortality, dialysis dependence at 28 and 90 days, ICU-free days, vasopressor-free days, ventilator-free days and RRT-free days; (2) inter-observer agreement; (3) correlation between VExUS, mVExUS, LUS, BIA and CFB; and (4) the relationship between changes in CFB, total ultrafiltration and ultrasound parameters on Day 1 and 3.

### Sample size calculation

We calculated the sample size based on a previous study with a 24% prevalence of venous congestion where the relative risk of mortality in patients with moderate to severe venous congestion by VExUS score was 2.03 [10]. Assuming a two-sided significance level of 0.05 and a power of 80% (β = 0.20), the required sample size was 98.

### Statistical analysis

Continuous data are reported as median (Q1, Q3) or mean (SD) and categorical data as number (%). Continuous variables were compared using the Mann–Whitney U test. Categorical variables were compared using Pearson’s chi-square test or Fisher’s exact test. LUS scores were treated as both continuous data and categorical data divided by median values at each timepoint. VExUS and mVExUS scores were planned *a priori* to be analysed as dichotomous variables: Grade 0−1 (normal/mild congestion) and Grade 2−3 (moderate/severe congestion) due to the expected low incidence of moderate to severe congestion in our cohort. Survival according to initial VExUS and mVExUS scores were depicted using Kaplan-Meier survival curves. The association between each parameter at Day 1 and Day 3 and mortality was assessed using Cox regression analysis, with multivariable analysis adjusted for the *a priori* specified SOFA score. Additional analyses were performed to assess the association between ultrasound markers as time-dependent variables and mortality through 90 days. No imputation was performed for missing values. Patients who died before the Day 3 assessment were treated as non-assessable for the Day 3 time point and were censored at their last known follow-up or date of death. Their Day 1 data were still included in the Day 1 baseline analyses. Results were expressed as hazard ratios (HRs) with 95% confidence intervals (CIs). For dialysis dependence at 28 and 90 days, a multinomial logistic regression was used to account for the competing risk of mortality. Results were expressed as relative risk ratio (RRR) with 95% CIs. For continuous secondary outcomes including ICU-, RRT-, ventilator- and vasopressor-free days, comparisons were performed using Wilcoxon rank-sum tests. Concordance index (C-statistics), net reclassification index (NRI) and integrated discrimination index (IDI) were analysed to compare if mVExUS improved the prognostic value over the traditional VExUS scores. For sensitivity analysis, we (1) analysed the results by treating VExUS scores and mVExUS scores as ordinal variables; (2) analysed the results by excluding patients with chronic liver disease; and (3) performed a landmark analysis in patients who survived and had Day 3 measurements. The relationship between ultrasound markers and changes in CFB and total ultrafiltration between Day 1 and 3 was assessed using generalized estimating equations (GEE) models with a logit link function and unstructured covariance matrix, which accounts for the repeated measures. The Spearman rank-order correlation coefficient was utilised to evaluate the association among different congestion surrogates. The degree of correlation was categorised as follows: 0.0–0.19 (very weak), 0.2–0.39 (weak), 0.4–0.59 (moderate), 0.60−0.79 (strong) and 0.8–1.0 (very strong). A two-sided p-value < 0.05 was considered statistically significant. All statistical analyses were performed using STATA (version 19.0, StataCorp LLC., College Station, TX, USA).

## Results

### Patient characteristics

A total of 100 eligible patients were enrolled (Supplementary Fig. S1). Fifty-eight percent of the study population was male, and 82% were medical ICU admissions, with a median age of 66 (interquartile range (IQR) 51−77) years and a median SOFA score of 13 (IQR 10−15). The majority (97%) required mechanical ventilation, and half (48%) had underlying CKD. The CFB from ICU admission to enrolment was 3.49% (IQR 1.35 %–7.01 %). The median time from ICU admission to CRRT initiation was 1 (IQR 0−3) day. The most common indications for CRRT initiation were oliguria (56%) and refractory acidosis (44%). CRRT was initiated due to refractory volume overload in 33% of patients. At the time of the Day 1 assessment, fluid removal had been initiated in 35 patients with a prescribed net ultrafiltration rate between 10−120 mL/hr (Table 1).

**Table 1:** Patient characteristics by VExUS grading at enrollment.

Variables	Overall (N = 100)	VExUS Grade 0−1 (n = 80)	VExUS Grade 2−3 (n = 20)	P-value
Demographics				
Age (years)	66 (51−77)	66 (50−77)	69 (56−78)	0.47
Male sex	58 (58%)	46 (57.5%)	12 (60%)	0.83
Medical ICU admission	82 (82%)	57 (83.7%)	15 (75%)	0.67
Comorbidities				
Chronic kidney disease	48 (48%)	35 (43.7%)	13 (65%)	0.08
eGFR baseline (mL/min/1.73 m^2^)	19.44 (13.8−32.7)	18.1 (12.8−32.7)	24.1 (18.1−30.5)	0.18
Hypertension	55 (55)	44 (55%)	11 (55%)	1
Diabetes mellitus	31 (31%)	26 (32.5%)	5 (25%)	0.51
Dyslipidemia	53 (53%)	42 (52.5%)	11 (55%)	0.84
Autoimmune disease	13 (13%)	12 (15%)	1 (5%)	0.23
Chronic lung disease	8 (8%)	5 (6.2%)	3 (15%)	0.19
Chronic liver disease	4 (4%)	4 (5%)	0	0.3
Peripheral artery disease	4 (4%)	2 (2.5%)	2 (10%)	0.12
Human immunodeficiency virus	2 (2%)	2 (2.5%)	0	0.47
Ischemic heart disease	21 (21%)	15 (18.7%)	6 (30%)	0.26
Malignancy	21 (21%)	18 (22.5%)	3 (15%)	0.46
Charlson comorbidity index	4 (2−7)	4 (2−7)	5.5 (3−7)	0.39
Mechanical ventilation	97 (97%)	77 (96.25%)	20 (100%)	0.37
Norepinephrine equivalent dose (mcg/kg/min)	0.22 (0.08−0.52)	0.2 (0.04−0.54)	0.23 (0.1−0.48)	0.7
Admission diagnosis				
Cardiovascular	19 (19%)	14 (17.5%)	5 (25%)	0.8
Sepsis	34 (34%)	27 (33.7%)	7 (35%)	
Gastrointestinal/hepatic	11 (11%)	8 (10%)	3 (15%)	
Respiratory	14 (14%)	11 (13.7%)	3 (15%)	
Neurologic	3 (3%)	3 (3.7%)	0	
Trauma	4 (4%)	4 (5%)	0	
Other	15 (15%)	13 (16.2%)	2 (10%)	
Sepsis	61 (61%)	47 (58.7%)	14 (70%)	0.35
APACHE II	21.5 (15.5−26)	21 (15−26)	22.5 (17.5−26.5)	0.23
SOFA	13 (10−15)	12.5 (9.5−15)	13.5 (10.5−15)	0.7
Time from ICU admission to CRRT initiation (days)	1 (0−3)	1 (0−2)	1 (0.5−5)	0.22
Indications for RRT				
Refractory acidosis	44 (44%)	37 (84.1%)	7 (15.9%)	0.36
Refractory volume overload	33 (33%)	27 (81.8%)	6 (18.2%)	0.75
Refractory hyperkalaemia	7 (7%)	7 (100%)	0	0.17
Anuria, oliguria	56 (56%)	46 (82.1%)	10 (17.8%)	0.54
Uraemia	17 (17%)	12 (70.6%)	5 (29.4%)	0.28
High BUN	33 (33%)	25 (75.7%)	8 (24.2%)	0.45
Echocardiographic parameters				
TAPSE (cm) (n = 60)	1.8 (1.5−2.1)	1.86 (1.59−2.1)	1.5 (1.16−1.7)	0.02
LVEF (%) (n = 73)	56 (38−66)	59 (37.5−65.5)	44 (38−66)	0.49
VTI (cm) (n = 58)	15.05 (12−20.2)	15.2 (12.3−20.6)	12.7 (9.3−17.2)	0.17
LV fractional shortening (%) (n = 75)	30.2 (18.9−36.9)	31.1 (20.8−37.1)	22.4 (18.5−34.4)	0.43
MV E:A ratio (n = 42)	0.96 (0.71−1.5)	0.88 (0.68−1.39)	1.6 (1.2−1.8)	0.05
E/Septal E’ (n = 62)	13.9 (9.2−17.2)	13.6 (8.8−16.7)	14.9 (10.2−17.3)	0.74
E/Lateral E’ (n = 60)	10.3 (7.05−13.8)	10.3 (7.1−12.6)	10.4 (6.9−16.3)	0.73
Tricuspid S’ vel (cm/sec) (n = 62)	11 (9−13.4)	11.2 (9.4−14.2)	9.7 (7.1−11.4)	0.04
RVSP (mmHg) (n = 50)	33.8 (28.3−42.4)	32.4 (28−40.7)	42.4 (29.6−52.5)	0.13
Central venous pressure (cmH_2_O) (n = 86)	14 (11−17)	13 (10.5−16)	16.5 (14−20)	0.004
Overhydration by BIA (L)	4.65 (2.3−6.8)	4 (2−6)	4 (2−8)	0.46
Cumulative fluid balance at enrolment (%)	3.49 (1.35−7.01)	3.65 (1.35−7.2)	2.05 (1.15−4.95)	0.13
Laboratory parameters				
Hb (g/dL)	8.5 (7.4−10.2)	8.35 (7.4−10.6)	8.75 (7.6−9.3)	0.78
WBC (/10^3^)	14 (7.2−20.7)	14.6 (7.9−22.1)	12.3 (6.3−16.2)	0.16
Neutrophils (/10^3^)	84.3 (76.1−90.5)	84 (76−90.5)	84.7 (83−91.2)	0.73
Lymphocytes (/10^3^)	7.05 (3.9−13.6)	7.05 (3.95−14)	7.7 (2.75−10.5)	0.52
Platelets (/10^3^/uL)	119.5 (70−206.5)	117.5 (73.5−215.5)	121.5 (47.5−168)	0.35
BUN (mg/dL)	57.5 (35.5−94.5)	59.5 (35.5−91)	47.5 (30.5−104)	0.89
Creatinine (mg/dL)	2.99 (1.84−3.95)	2.91 (1.8−3.95)	3.09 (2.06−4.02)	0.37
Sodium (mEq/L)	138 (135−143)	138 (135−143)	138.5 (134.5−144.5)	0.74
Potassium (mEq/L)	4.15 (3.75−4.85)	4 (3.8−4.9)	4.2 (3.45−4.55)	0.54
Chloride (mEq/L)	101 (97−106)	101 (97−106)	101 (97−104)	0.76
Carbon dioxide (mEq/L)	16 (11.5−20)	16 (11−19.5)	20 (14−22)	0.14
Lactate (mg/dL)	5.15 (2−11)	5.35 (10.6)	4.15 (1.6−13.1)	0.94
Urinary NGAL (ng/mL) (n = 62)	1809 (267−4327.6)	1970 (261−4327)	1544 (599−1964)	0.97
NT-proBNP (pg/mL) (n = 95)	14661 (3905−35000)	14943 (2727−35000)	12396 (9081−34811)	0.36

Abbreviations: *AKI* acute kidney injury, *APACHE II* Acute Physiology and Chronic Health Evaluation, *BIA* Bioelectrical Impedance Analysis, *BUN* blood urea nitrogen, *Cr* creatinine, *eGFR* estimated glomerular filtration rate, *Hb* Hemoglobin, *ICU* intensive care unit, *IQR* interquartile range, *IVC* inferior vena cava, *LVEF* left ventricular ejection fraction, *NGAL* neutrophil gelatinase-associated lipocalin, *NT-proBNP* N-terminal pro B-type natriuretic peptide, *SD* standard deviation, *SOFA* sequential organ failure assessment, *TAPSE* tricuspid annulus plane systolic exertion, *VTI* velocity time integral, *WBC* white blood cell.

### Ultrasound assessments and reproducibility

A total of 180 ultrasound assessments were performed: 100 at enrolment and 80 on Day 3. Image acquisition success rates were overall high, ranging from 92.7 %–93.8 % for intrarenal venous Doppler, 96.1–99.2 % for hepatic vein Doppler and 98–100 % for portal vein Doppler. At least two sites were assessable for VExUS grading in 98% of assessments (Supplementary Table S1). Inter-rater agreement was good, with a weighted Cohen’s kappa of 0.86. Similar agreement was observed using PABAK (0.72) and Gwet’s AC1 (0.78). Hepatic vein (weighted kappa 0.94) and intrarenal vein (weighted kappa 0.92) assessments demonstrated excellent reliability, whereas portal vein assessments had moderate agreement (weighted kappa 0.52). (Supplementary Table S2).

### Prevalence of venous and pulmonary congestion

**Fig. 1:**
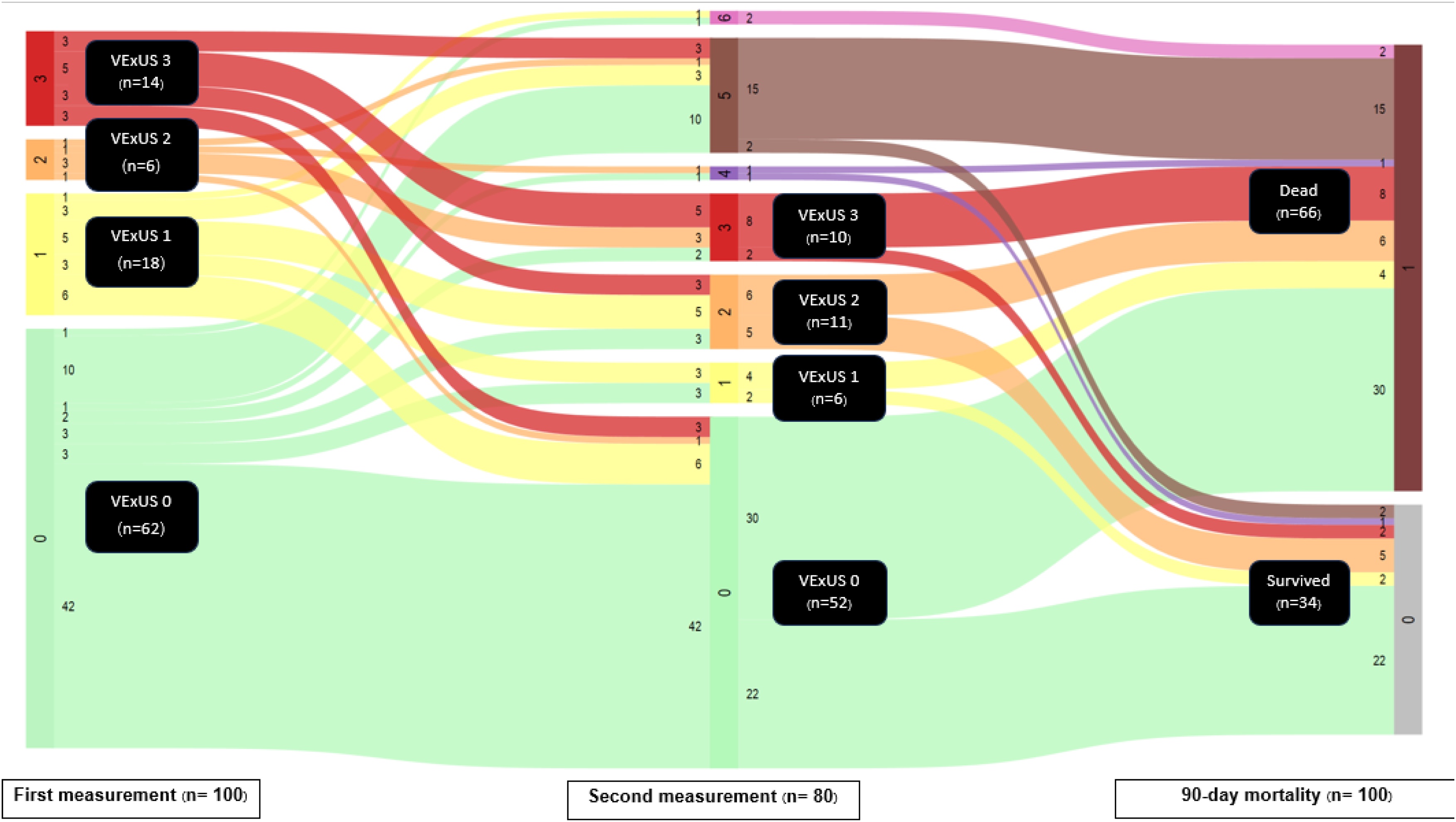
Alluvial diagram showing the evolution of VExUS grading between Day 1 and 3 and mortality at 90 days. Green color—VExUS = 0. Yellow color—VExUS = 1. Orange color—VExUS = 2. Red color. VExUS = 3. Purple – patients who received ECMO. Brown – patients who died. Pink – patients who discontinued CRRT.

Alluvial diagrams illustrate changes in VExUS and mVExUS grading over time (Fig. 1, Supplementary Fig. S2). At enrolment, moderate-to-severe venous congestion (Grade 2−3) was identified in 20% of patients using VExUS criteria and in 35% of patients using mVExUS criteria. Moderate-to-severe congestion patterns were documented in 54% of hepatic vein assessments, 62% of portal vein assessments and 63% of intrarenal vein assessments. The median maximal IVC diameter was 1.80 cm (IQR 1.56–2.21 cm), and the median PPF was 34.2% (IQR 24.3 %–41.9 %). At 72 h, the prevalence of VExUS and mVExUS Grade 2–3 was 26% and 41% of reassessed patients, respectively. The IVC diameter significantly increased to 1.82 cm (IQR 1.33–2.12 cm) at 72 h (Supplementary Table S3).

Compared with VExUS Grade 0−1, patients with VExUS Grade 2−3 at enrolment were more likely to have higher CVP, lower TAPSE, lower tricuspid S’ velocity and higher MV E:A ratio values. **(**Table 1**)** Similarly, patients with mVExUS Grade 2−3 at enrolment had lower TAPSE, tricuspid S’ velocity and LVOT VTI and higher RVSP and CVP values compared with patients who had mVExUS Grade 0−1 (Supplementary Table S4).

The median LUS scores were 4 (1–9) at enrolment and 3 (1–9) at 72 h.

### Relationship between ultrasound parameters and fluid balance

**Fig. 2:**
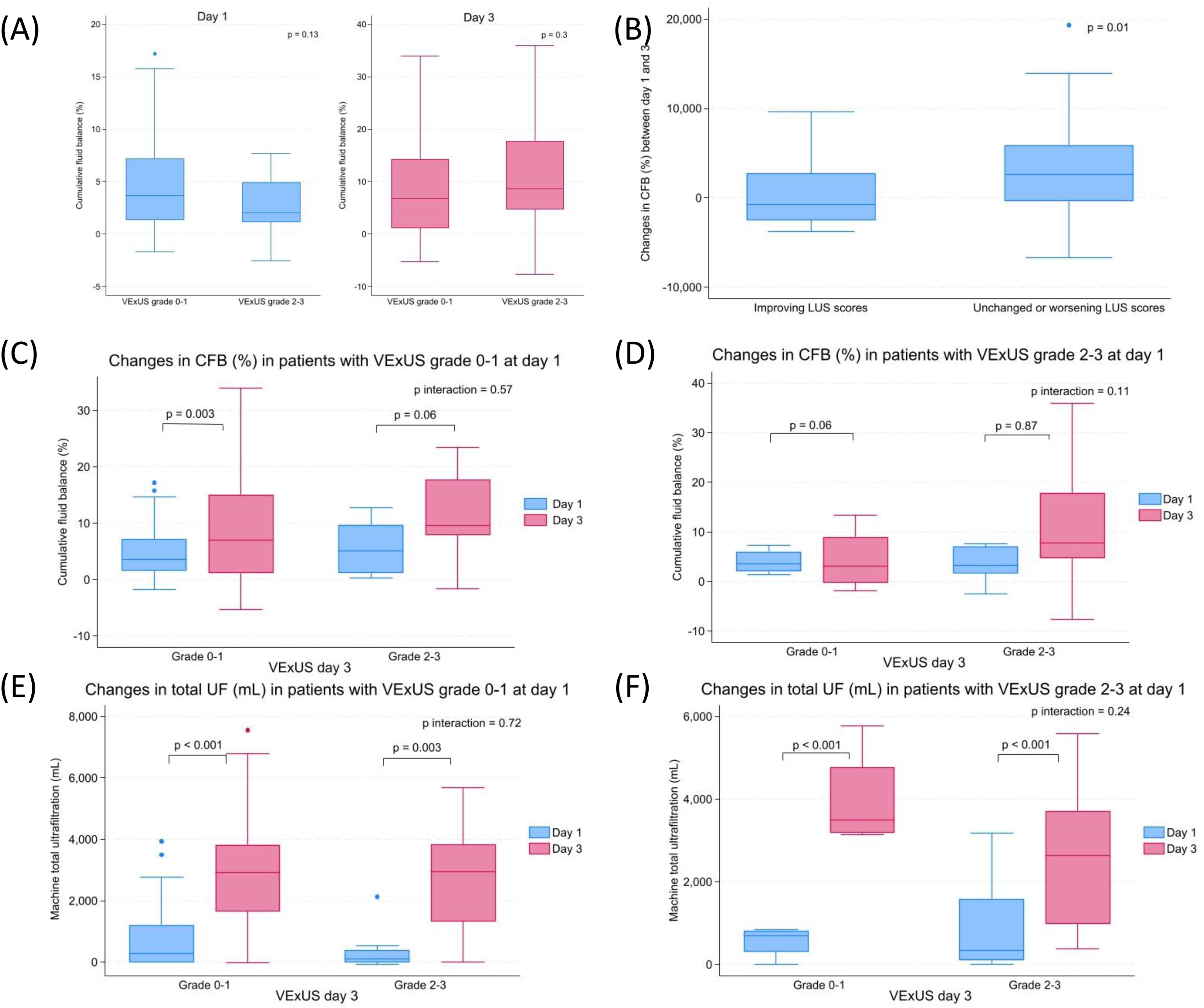
Boxplots showing changes in cumulative fluid balance (%) and machine total ultrafiltration (mL) between Day 1 and 3 stratified by venous excess ultrasound score (VExUS) grade and lung ultrasound (LUS). (A) Comparison of cumulative fluid balance (%) between VExUS grade 0−1 and 2−3 on Day 1 and 3. (B) Comparison of changes in cumulative fluid balance (%) between Day 1 and 3 in patients with worsening or unchanged LUS scores and patients with improved LUS scores. (C–F) Changes in cumulative fluid balance (%) from Day 1 to Day 3 for patients with VExUS Grade 0–1 (C) and Grade 2–3 (D) at enrollment. (E–F) Changes in total ultrafiltration (mL) from Day 1 to Day 3 in patients with VExUS Grade 0–1 (E) and Grade 2–3 (F) at enrolment. Brackets indicate pairwise p-values and reported interaction p-values compare time-by-VExUS group effects; blue = day 1, pink = day 3.

There were no differences in CFB at the initial ultrasound assessment (p = 0.13) and on Day 3 (p = 0.3) between patients with VExUS Grade 0−1 and 2−3 (Fig. 2A). For patients with VExUS Grade 0−1 at Day 1 (Fig. 2C, 2E), CFB significantly increased in those who remained Grade 0−1 (p = 0.003) and trended to increase in those who progressed to Grade 2−3 (p = 0.06); however, there was no significant difference in the change in CFB between these subgroups (p interaction = 0.57). In both of these subgroups, total ultrafiltration significantly increased from Day 1 to Day 3, with no significant difference in the magnitude of this increase between groups (p interaction = 0.72). In contrast, for patients with VExUS Grade 2−3 at Day 1 **(**Fig. 2D, 2F), CFB demonstrated a trend to decrease in those who improved to Grade 0−1 (p = 0.06), but remained unchanged in those who stayed at Grade 2−3 (p = 0.87). Similarly, while total ultrafiltration significantly increased from Day 1 to Day 3 in both of these subgroups (p < 0.001 for both), there was no significant difference in the magnitude of this increase between those who improved their VExUS grade and those who remained at Grade 2−3 (p interaction = 0.24). Exploratory analyses utilising the mVExUS criteria yielded similar trends regarding fluid removal and score trajectory (Supplementary Figure S4). Regarding pulmonary congestion, patients whose LUS scores remained the same or worsened (i.e., increased LUS scores) demonstrated a significant increase in CFB between Day 1 and 3 compared to those whose LUS improved (i.e. decreased LUS scores) (p = 0.01) (Fig. 2B).

### Association with the principal clinical endpoint

Compared with 90-day survivors, non-survivors were older (72 vs. 56 years; p = 0.01), received a higher norepinephrine equivalent dose (0.28 vs. 0.11 mcg/kg/min; p = 0.01) and had a higher SOFA score (14 vs 12; p = 0.04) (Supplementary Table S5).

**Fig. 3:**
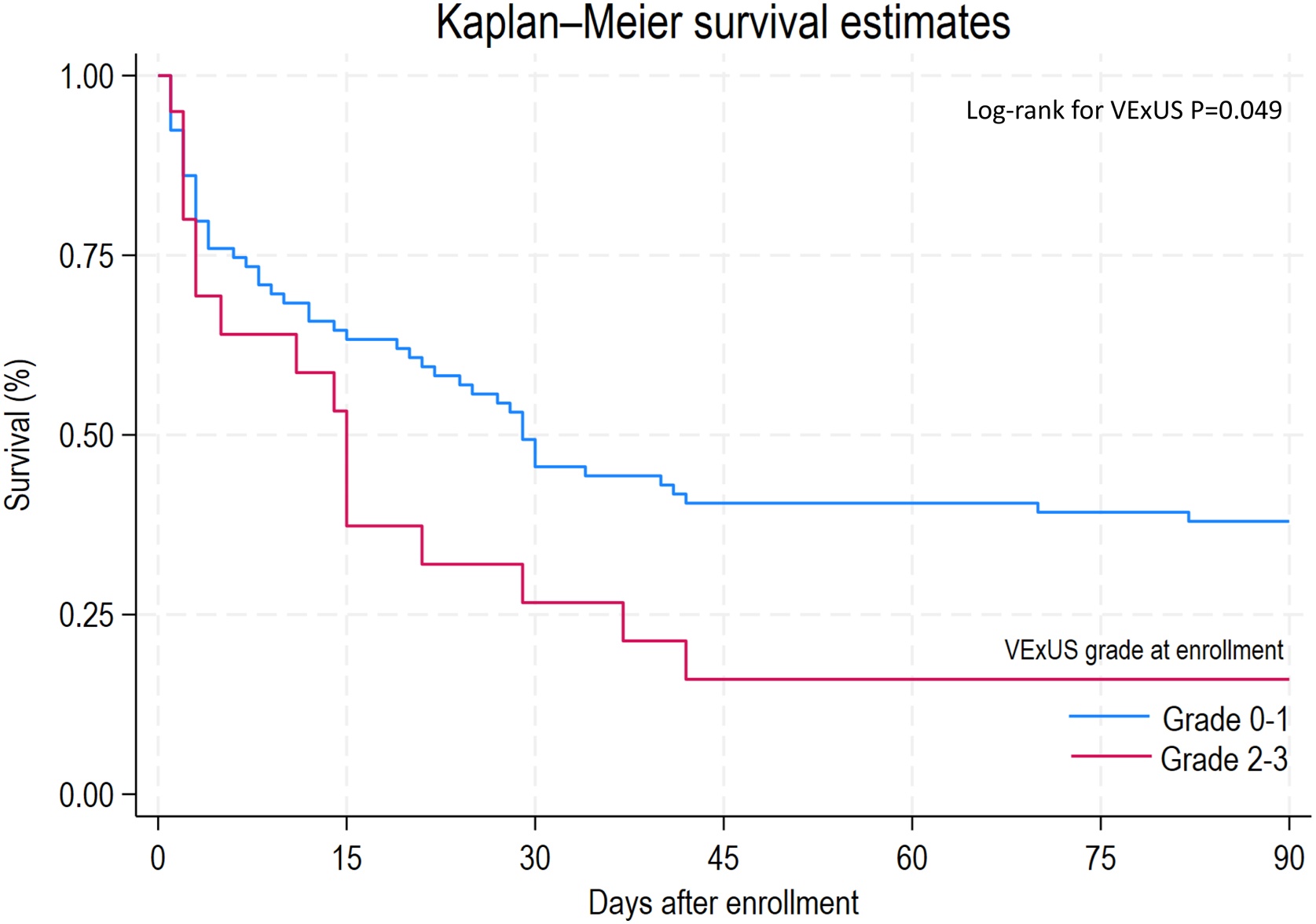
Kaplan-Meier curves comparing 90-day survival between patients with VExUS Grade 0-1 and 2-3 at enrolment.

At 90 days, the Kaplan–Meier survival analyses showed lower survival in the baseline VExUS Grade 2−3 group (Log rank p = 0.049) (Fig. 3) and mVExUS Grade 2–3 group (Log rank p = 0.01) (Supplementary Figure 3) compared with their respective 0−1 groups. In univariable Cox regression analysis, VExUS Grade 2−3 at enrollment showed a trend toward higher 90-day mortality compared to those with Grade 0−1 (HR 1.73; 95% CI 0.98−3.06, p = 0.05), and mVExUS Grade 2−3 was significantly associated with 90-day mortality (HR 1.78; 95% CI 1.08–2.91, p = 0.024). On multivariable Cox-regression analysis adjusting for the initial SOFA score, mVExUS grade 2–3 was independently associated with 90-day mortality (adjusted HR 1.66; 95% CI 1.01–2.75, p = 0.04) (Table 2).

**Table 2:** Cox proportional hazard analysis of associations between variables at enrolment and 90-day mortality.

Variables	HR (95% CI)	P value	aHR (95% CI)*	P value
Age	1.01 (0.99−1.02)	0.06		
Male sex	0.94 (0.57−1.54)	0.81		
Norepinephrine equivalent dose (mcg/kg/min)	1.49 (1.01−2.2)	0.04		
SOFA score	1.05 (0.99−1.12)	0.08	1.04 (0.98−1.11)	0.16
Lactate (mmol/L)	1.05 (1.01−1.09)	0.01		
LUS score	1.007 (0.96−1.05)	0.73		
CVP (cmH2O)	1.008 (0.96−1.05)	0.74		
OH by BIA (per 1 L)	1.04 (0.96−1.12)	0.30		
NT-proBNP	1 (1)	0.57		
CFB (per 1 L)	1.01 (0.93−1.1)	0.73		
VExUS				
Grade 0−1	Reference			
Grade 2−3	1.73 (0.98−3.06)	0.05		
mVExUS				
Grade 0−1	Reference		Reference	
Grade 2−3	1.77 (1.07−2.91)	0.02	1.66 (1.007−2.75)	0.04

Abbreviations: BIA, bioimpedance analysis, CFB, cumulative fluid balance; CI, confidence interval; CVP, central venous pressure; HR, hazard ratio; mVExUS, modified venous excess ultrasound; NT-proBNP N-terminal pro B-type natriuretic peptide; OH, overhydration; SOFA, Sequential Organ Failure Assessment; VExUS, venous excess ultrasound.

* p < 0.05.

In the time-updated Cox models adjusting for baseline SOFA score, hepatic vein Doppler Grade 2−3 (aHR 1.78; 95% CI 1.05–3.01, p = 0.01) and LUS scores (aHR 1.04; 95% CI 1.001–1.08, p = 0.04) were independently associated with 90-day mortality. Time-updated mVExUS Grade 2−3 showed a marginally significant association (HR 1.38; 95% CI 0.94–2.01, p = 0.09) and time-updated VExUS Grade 2−3 did not show any association with 90-day mortality (HR 1.13; 95% CI 0.65–1.95, p = 0.65) (Supplementary Table S6).

To evaluate whether the exploratory mVExUS criteria improved prognostic accuracy over the traditional VExUS score, we performed comparative performance testing (Supplementary Table S7). mVExUS demonstrated a modest improvement in predictive performance for 90-day mortality compared with VExUS (C-statistic 0.65 vs 0.60). However, this difference did not reach statistical significance for discrimination (C-statistic) or reclassification (NRI) and was marginally significant for IDI (p = 0.049).

### Association with secondary endpoints

There were no significant differences in dialysis dependence at 28 and 90 days between higher and lower baseline VExUS and mVExUS scores. Patients with VExUS Grade 2−3 showed a trend towards fewer ICU-free days (p = 0.08) and vasopressor-free days (p = 0.06). Patients with mVExUS Grade 2−3 at enrolment had higher 28-day mortality (p = 0.045), 90-day mortality (p = 0.024) and fewer vasopressor-free days (p = 0.04) compared with mVExUS Grade 0−1. LUS scores at baseline were associated with increased RRT dependence at day 90 (RR 1.06; 95% CI 1.02–1.09, p = 0.002) (Supplementary Table S8 and S9).

### Sensitivity analyses

When treated as ordinal variables, VExUS grade 2 (HR 2.72; 95% CI 1.04–7.1, p = 0.04) and mVExUS grade 3 (HR 1.88; 95% CI 0.96–3.67, p = 0.06) were associated with 90-day mortality (Supplementary Table S10). A sensitivity analysis excluding 4 patients with chronic liver disease showed a trend towards an increased risk of 90-day mortality in patients with baseline VExUS Grade 2−3 (aHR 1.67; 95% CI 0.95–2.95, p = 0.08) and a significantly increased risk of 90-day mortality in patients with baseline mVExUS Grade 2−3 (aHR 1.77; 95% CI 1.06–2.95, p = 0.02) (Supplementary Table S11). In a prespecified landmark analysis, VExUS and mVExUS scores on Day 3 were not associated with secondary clinical outcomes. Higher LUS scores at Day 3 were associated with an increased risk of 28-day mortality (p = 0.001) and 90-day mortality (p < 0.001), as well as fewer ICU-free days (p = 0.01), vasopressor-free days (p = 0.01) and ventilator-free days (p = 0.008) (Supplementary Table S12).

### Bioimpedance analysis

There were no differences in overhydration as reported by BIA between those with VExUS or mVExUS Grade 0−1 and 2−3. BIA showed a moderate correlation with CFB (r = 0.50, p = <0.001, n = 94) but no correlations with VExUS and mVExUS scores. Overhydration by BIA was not associated with 90-day mortality (HR 1.04; 95% CI 0.96−1.12, p = 0.3).

### Correlation analyses

A moderate correlation was noted between CVP and intrarenal vein Doppler flow (r = 0.57, p < 0.001, n = 86). Conversely, TAPSE, a marker of right ventricular function, demonstrated negative correlations with hepatic vein Doppler flow (r = −0.38, p = 0.001, n = 60), intrarenal vein Doppler flow (r = −0.33, p = 0.003, n = 60), NT-proBNP (r = −0.43, p = 0.001, n = 59) and mVExUS (r = −0.36, p = 0.005, n = 60). While LUS showed a statistically significant moderate correlation with hepatic vein Doppler flow (r = 0.40, p < 0.001, n = 100), its relationship with CFB was weak and did not reach statistical significance (r = −0.18, p = 0.06, n = 100) (Supplementary Table S13).

## Discussion

In this prospective observational study of serial ultrasound assessments in 100 critically ill patients with AKI receiving CRRT, moderate to severe venous congestion was present in 20% (VExUS) and 35% (mVExUS) of patients at enrolment. Patients with venous congestion at baseline were more likely to exhibit signs of right ventricular dysfunction, namely lower TAPSE and tricuspid S’ velocity. Regarding our principal clinical endpoint, patients with higher baseline VExUS and mVExUS scores had lower 90-day survival rates. In the time-updated analyses, persistent hepatic vein abnormalities (Grade 2−3) and higher LUS scores at 72 h were associated with 90-day mortality. Notably, neither VExUS nor mVExUS grades were associated with kidney endpoints, specifically dialysis dependence at 28 and 90 days. Finally, we observed that the evolution of LUS scores correlated with changes in CFB.

Our cohort consisted predominantly of medical patients with high severity of critical illness. Despite this, CRRT was initiated due to refractory volume overload in only one-third of the cohort, as reflected by zero targets of prescribed net ultrafiltration rates in 35% of the patients. This may partly explain the lower prevalence of venous congestion, compared to that previously reported in general ICU populations and severe AKI patients [10,[21], [22], [23]]. We observed an association between moderate to severe venous congestion and increased risk of mortality, which underscores the detrimental impact of FAS on patient outcomes [2]. It is important to recognise that VExUS reflects the interaction between cardiac function, filling pressures and volume status. Previous studies have demonstrated a strong positive correlation between VExUS grading and mean systemic filling pressure, right atrial pressure and pulmonary capillary wedge pressures [24,25]. However, hepatic vein Doppler pattern can be influenced by right ventricular contraction and tricuspid valve movement. Cirrhosis can alter portal vein waveform patterns due to decreased hepatic compliance and/or portal hypertension [26]. Cardiac function and local renal haemodynamics can also influence intrarenal venous flow patterns [27]. Furthermore, moderate inter-rater agreement for portal vein Doppler flow may be explained by physiological variability between each assessment and operator dependence. The observed increase in maximal IVC diameter between Day 1 and Day 3, despite ongoing CRRT, may reflect dynamic shifts in intravascular volume status, changes in ventilatory settings, or cardiac pressures. However, the overall VExUS grading aims to mitigate this by integrating multiple Doppler parameters to provide a comprehensive assessment of systemic congestion.

Similar to findings by Wong et al. [12], we observed that higher VExUS scores were correlated with markers of RV dysfunction. Therefore, Doppler-based ultrasound markers may serve as prognostic indicators of right ventricular strain or systemic venous congestion, both of which are associated with mortality risk. We have not demonstrated differences in AKI resolution between patients with and without moderate-to-severe venous congestion, which may be explained by the overall severity of the cohort and competing risk of mortality.

Recognising that IVC diameters are confounded by various factors and may vary with Asian demographics [18,26], we evaluated an alternative mVExUS criteria. Although the prevalence of venous congestion increased using mVExUS grading compared to traditional VExUS criteria, comparative performance testing showed that mVExUS modestly improved the ability to predict 90-day mortality and only reached statistical significance for IDI. Therefore, mVExUS should be viewed as an exploratory, hypothesis-generating tool that requires formal external validation before broader clinical application.

Our study explored the dynamic relationship between POCUS markers and fluid management. Point-of-care ultrasound has been proposed as a useful adjunct in assessing volume status and guiding volume removal by CRRT. Previous studies have shown that VExUS-guided therapy was significantly associated with AKI resolution and successful decongestion in cardiorenal syndrome [11,28]. In a previous study of ESKD patients, VExUS scores improved after fluid removal by intermittent dialysis [12]. Similarly, the trajectory of VExUS grades over time was related to fluid management. Among patients with moderate-to-severe congestion at Day 1, those who subsequently improved to Grade 0−1 experienced a trend towards reduced CFB and were able to achieve substantial total ultrafiltration from Day 1 to Day 3. This contrasts with those who remained in Grade 2−3, who did not show significant improvement in fluid balance despite ongoing fluid removal. Conversely, among patients starting with VExUS Grade 0−1, those who worsened to Grade 2−3 still underwent significant fluid removal, yet their CFB also increased, highlighting the complexity of fluid dynamics and the role of factors beyond fluid removal alone in critical illness.

Lung ultrasound can be used to detect vertical “B-lines,” which represent extravascular lung water. In ESKD, B-lines are more sensitive for detection of lung congestion than auscultation [5]. Studies of LUS in patients with AKI have been limited, but some demonstrate that B-lines correlate with hypoxemia, weaning failure and weight gain [13,29]. LUS-guided fluid removal in AKI has been associated with reduced intradialytic events [30]. Our study showed associations between higher LUS scores and worse outcomes. Changes in CFB correlated with dynamic changes in LUS scores. This suggests that serial VExUS and LUS tracking may offer a valuable bedside monitor to assess whether patients respond to ultrafiltration during CRRT.

While our study showed a moderate correlation between BIA and CFB, overhydration by BIA was not shown to be associated with mortality. VExUS and LUS scores were not significantly correlated with CFB in our cohort. This is consistent with published studies and may be explained by factors such as endothelial leakage, vasodilatation and cardiac dysfunction in critically ill patients [[31], [32], [33]], implying that intravascular volume status by VExUS might not directly reflect total body water.

## Strengths and limitations

This prospective study provides evidence supporting the use of serial multimodal POCUS techniques in general ICU patients with AKI receiving CRRT. VExUS scores showed high interobserver agreements, similar to previous studies [34,35]. Both VExUS and LUS scores correlated with clinically relevant outcomes. However, several limitations must be acknowledged. First, as a prospective observational study, our design precludes the inference of causality. While we identified significant associations between markers of congestion and patient outcomes, we cannot conclude that congestion directly causes mortality or that targeting these markers will improve outcomes. Our findings primarily support the prognostic utility of these markers of severe, persistent haemodynamic and cardiopulmonary stress. Second, despite adjusting for baseline SOFA score, our models are susceptible to residual confounding and other unadjusted variables owing to limited sample size. The critically ill nature of our cohort means numerous dynamic factors, such as vasopressor doses, ventilatory settings (e.g., positive end-expiratory pressure), specific underlying cardiac function (e.g., RV dysfunction and other unmeasured comorbidities), could influence both congestion markers and outcomes. In addition, comprehensive echocardiography was not performed in all cases. Detailed cardiac function assessment can be valuable in differentiating between cardiac-driven and volume-driven congestion. Third, our study involved multiple comparisons across outcomes, which should be considered exploratory endpoints. We did not formally adjust for multiplicity, meaning some findings should be interpreted with caution regarding type I error. Moreover, the longitudinal analyses are subject to informative censoring, as patients who died before the Day 3 assessment were excluded from time-updated analyses and landmark analyses at Day 3, potentially biasing associations towards a healthier surviving population. Fourth, the modest power, coupled with the dichotomisation of VExUS grading (for clinical interpretability) may have limited our ability to detect more subtle associations in a graded manner. The mVExUS construct is an exploratory, hypothesis-generating adaptation, with IVC thresholds adopted from existing literature and not independently validated within our cohort. While it demonstrated associations with mortality in our population, external validation in diverse populations is required. Fifth, our study was conducted at a single centre, to which the results need to be externally validated in diverse populations. The exclusion of patients with conditions such as cirrhosis, tricuspid regurgitation, structural kidney diseases, ARDS or those who received ECMO prohibits the application of our findings to these complex CRRT populations [36]. Finally, while systemic congestion was associated with worse outcomes in AKI on CRRT, the optimal fluid management strategy often remains unclear. Patients with fluid intolerance might respond to fluid administration, while patients who are fluid responsive might not necessarily be fluid tolerant [37,38]. Although volume status assessment is fundamental, this must be accompanied by an optimal fluid management strategy which can be feasibly incorporated into routine patient care and allow for repeated assessments.

## Conclusion

In patients with severe AKI requiring CRRT, systemic venous and pulmonary congestion, as assessed by VExUS and LUS were associated with an increased risk of 90-day mortality. Longitudinal changes in LUS in patients during CRRT correlated with CFB changes. These findings suggest that serial multiorgan POCUS may serve as a valuable prognostic tool. Further studies are encouraged to investigate its role in guiding individualised fluid management in this population and its impact on patient-centred outcomes including mortality and AKI resolution.

## Authors' contributions

TT: Conceptualization, formal analysis, data curation, methodology, visualization, writing - original draft.

AW: Data curation, Writing - review & editing.

SP: Investigation, Writing - review & editing.

YA: Investigation, Writing - review & editing.

MO: Methodology, Writing - review & editing.

NS: Investigation, Resources, Methodology, Supervision, Writing - review & editing.

NL: Conceptualization, formal analysis, data curation, funding acquisition, methodology, visualization, writing - original draft.

## Consent for publication

Not applicable.

## Ethics approval and consent to participate

The study was approved by the Faculty of Medicine, Chulalongkorn University ethics committee (0899/66). All participants or legal representatives provided written consent.

## Declaration of Generative AI and AI-assisted technologies in the writing process

None.

## Funding

Excellence Centre for Critical Care Nephrology, King Chulalongkorn Memorial Hospital, Bangkok, Thailand.

## Availability of data and materials

The datasets used and/or analysed during the current study are available from the corresponding author on reasonable request.

## Data availability

The data that has been used is confidential.

Data will be made available on request.

## Declaration of competing interest

The authors declared no competing interests.

## Footnotes

Appendix A Supplementary material related to this article can be found, in the online version, at doi:https://doi.org/10.1016/j.aicoj.2026.100129.
