# Supplementary material for: Venous excess and lung ultrasound during continuous renal replacement therapy in critically ill patients (VExLUS-RRT): a prospective observational study
Source: Ann Intensive Care. 2026 Jul 30;16:100129. doi: 10.1016/j.aicoj.2026.100129 (PMC13475691; doi:10.1016/j.aicoj.2026.100129)
Supplement: Supplementary file 1 [file mmc1.docx]

**Supplementary material**

| **Appendix** | **Page** |
| --- | --- |
| **Additional methods** | **2** |
| **Supplementary Figure 1: Flow diagram** | **6** |
| **Supplementary Figure 2: Alluvial diagram showing the profiles of evolution of mVExUS grading during the first 72 h in ICU and mortality at 90 days** | **7** |
| **Supplementary Figure 3: Kaplan-Meier curves comparing 90-day survival between patients with mVExUS grade 0-1 and 2-3 at enrolment** | **8** |
| **Supplementary Figure 4: Boxplots showing cumulative fluid balance (%) and machine total ultrafiltration (mL) between Day 1 and 3 and the evolution of mVExUS grading stratified by the initial mVExUS grade** | **9** |
| **Supplementary Table 1: The success rates of each ultrasound measurement** | **10** |
| **Supplementary Table 2: Interobserver agreement of ultrasound assessment** | **11** |
| **Supplementary Table 3:** **Evolution of ultrasound parameters from enrolment to Day 3** | **12** |
| **Supplementary Table 4: Patient characteristics by mVExUS grading at enrolment** | **13** |
| **Supplementary Table 5:** **Patient characteristics and parameters at enrolment in relationship with 90-day mortality** | **16** |
| **Supplementary Table 6:** **The association between ultrasound markers as time-dependent variables and mortality through 90 days** | **19** |
| **Supplementary Table 7: Comparison of the C-statistics, NRI, and IDI of VExUS versus mVExUS in predicting 90-day mortality** | **20** |
| **Supplementary Table 8: Association between VExUS and mVExUS grading at baseline and secondary outcomes** | **21** |
| **Supplementary Table 9:** **Association between LUS scores on Day 1 and 3 and outcomes** | **22** |
| **Supplementary Table 10: Sensitivity analysis: univariable and multivariable analysis of VExUS and mVExUS scores on Day 1 as ordinal variables and 90-day mortality** | **23** |
| **Supplementary Table 11: Sensitivity analysis: Cox proportional hazard analysis of associations between variables at enrolment and 90-day mortality in patients without chronic liver disease (n=96)** | **24** |
| **Supplementary Table 12:** **Sensitivity analysis: association between VExUS and mVExUS grading on Day 3 and secondary outcomes** | **25** |
| **Supplementary Table 13: Spearman’s Correlation between parameters to assess fluid status** | **26** |
| **References** | **27** |

**Additional methods**

**Ultrasound assessments**

Ultrasound studies were performed at the bedside using an ultrasound machine with a phased array transducer and/or a convex array transducer by investigators trained in critical care ultrasound (TT, AW or NL).

**Venous Excess Ultrasound [4]**

Patients were positioned in a dorsal decubitus position during the examination with the head of the bed at 0 to 30°. Concurrent electrocardiogram was obtained to precisely identify the phases of the cardiac cycle. The measurements were performed at the end

of expiration.

Portal vein Doppler assessment was performed by positioning the probe to obtain a right coronal view of the liver to identify the hepatic hilum where the main portal vein enters the liver. Pulse-wave ultrasound was used to record the peak (VMax) and the nadir velocities (VMin) during the cardiac cycle.

From the same view, pulsed wave Doppler waveform recording of the hepatic venous flow was obtained by interrogating the right hepatic vein during three consecutive cardiac cycles, and the pattern between the systolic and diastolic velocity (S/D) was observed.

Intra-renal Doppler assessment was obtained with a coronal longitudinal view. After identifying vessels using color Doppler, pulsed wave Doppler waveform at the corticomedullary junction was obtained in the middle segment during three consecutive cardiac cycles.

The dimensions of the inferior vena cava were measured in its intra-hepatic portion using a sub-xiphoid window to obtain both longitudinal and transverse views. The collapsibility index was measured 2 cm after the hepatic vein junction using M-mode on the longitudinal view. An IVC with a collapsibility index of less than 50% or distensibility index of less than 18% was considered plethoric.

**VExUS score**

1. Hepatic vein (HV): by pulsed wave doppler

Grade 1 = normal S>D

Grade 2 = S<D with antegrade S

Grade 3: S flat or inverted or biphasic trace

2. Portal vein (PV) doppler

The portal vein pulsatility fraction (PPF) was calculated as follows:

PPF (%) = 100 [(VMax - VMin))⁄(VMax]

Grade 1 = <0.3 PPF

Grade 2 = 0.3-0.49 PPF

Grade 3 = 0.5-1.0 PPF

3. Intra renal vein (IRV) doppler

Grade 1 = continuous waveform

Grade 2 = Biphasic distinct S, D waves

Grade 3 = Monophasic D-only below baseline

Venous Excess Ultrasound Score (VExUS) is categorised into 4 grades.

Grade 0: IVC < 2 cm, whatever HV/PV/IRV pattern

Grade 1: IVC ≥ 2 cm but normal HV/PV/IRV pattern

Grade 2: IVC ≥ 2 cm with one grade 3 flow pattern abnormalities in HV/PV/IRV

Grade 3: IVC ≥ 2 cm with two grade 3 flow pattern abnormalities in HV/PV/IRV

Modified Venous Excess Ultrasound Score (mVExUS) is categorised into 4 grades.

Grade 0: IVC collapsibility >50% or distensibility >18%, whatever HV/PV/IRV pattern

Grade 1: IVC collapsibility >50% or distensibility >18%, but normal HV/PV/IRV pattern

Grade 2: IVC collapsibility >50% or distensibility >18%, with one grade 3 flow pattern abnormalities in HV/PV/IRV

Grade 3: IVC collapsibility >50% or distensibility >18%, with two grade 3 flow pattern abnormalities in HV/PV/IRV

**VExUS Classification** Adapted from **[1]**

**Lung ultrasound [28]**

Lung ultrasound was performed using a curvilinear ultrasound probe or a phased Array probe at 6 different areas.

Sites:

1. Anterior chest (R1 and L1) between mid-clavicular line at 2^nd^ intercostal space of right and left lung, respectively.
2. Lateral chest (R2 and L2) at mid axillary line around the 6-7^th^ intercostal space.
3. Posterior chest (R3 and L3) intersection of the posterior axillary line and a rib space between 10^th^ and 12^th^ ribs.

B-lines were identified as following:

Appear ray-like, hyperechoic, and vertical

Originate from pleural lines

Move with lung sliding to confirm fluid overload.

Lung ultrasound is classified into 4 grades:

Grade 0: A lines

Grade 1: less than 3 B-lines

Grade 2: less than 50% of B-lines

Grade 3: more than 50% of B-lines

The presence of pleural effusion for each hemithorax adding one more point to final score.

Total maximum lung ultrasound score = 20 points.

**The Lung Ultrasound Score ranges from 0 to 20 points** Adapted from **[1]**

**Supplementary Figure 1: Flow diagram**

**Supplementary Figure 2: Alluvial diagram showing the profiles of evolution of mVExUS grading during the first 72 h in ICU and mortality at 90 days**

Green color—mVExUS =0. Yellow color—mVExUS = 1. Orange color—mVExUS =2. Red color—mVExUS =3. Purple – patients who received ECMO. Brown – patients who died. Pink – patients who discontinued CRRT.

**Supplementary Figure 3: Kaplan-Meier curves comparing 90-day survival between patients with mVExUS grade 0-1 and 2-3 at enrolment**

Log-rank p = 0.01

**Supplementary Figure 4: Boxplots showing cumulative fluid balance (%) and machine total ultrafiltration (mL) between Day 1 and 3 and the evolution of mVExUS grading stratified by the initial mVExUS grade**

(A) Comparison of cumulative fluid balance (%) between mVExUS grade 0-1 and 2-3 on day 1 and 3. (B-C) Changes in cumulative fluid balance (%) from day 1 to day 3 for patients with mVExUS grade 0–1 (B) and grade 2–3 (C) at enrollment. (D–E) Changes in total ultrafiltration (mL) from day 1 to day 3 for patients with mVExUS grade 0–1 (D) and grade 2–3 (E) at enrolment. Brackets indicate pairwise p-values and reported interaction p-values compare time-by-mVExUS group effects; blue = day 1, pink = day 3.

| (A)   | |
| --- | --- |
| (B)   | (C)   |
| (D)   | (E)   |

**Supplementary Table 1: The success rates of each ultrasound measurement**

| **Operator 1** | **First assessment (N=100)** | **Second assessment (N=80)** | **All assessments**  **(N=180)** |
| --- | --- | --- | --- |
| Renal venous Doppler | 92% | 96.25% | 93.8% |
| Portal vein Doppler | 100% | 100% | 100% |
| Hepatic vein Doppler | 97% | 95% | 96.1% |
| VExUS * (Success in at least 2 sites) | 100% | 97.5% | 98.8% |
| **Operator 2** | **First assessment** | **Second assessment** | **All assessments** |
| Renal venous Doppler | 95% | 90.1% | 92.7% |
| Portal vein Doppler | 98% | 98.3% | 98.4% |
| Hepatic vein Doppler | 98% | 100% | 99.2% |
| VExUS * (Success in at least 2 sites) | 98% | 98.3% | 98.3% |

**Supplementary Table 2: Interobserver agreement of ultrasound assessment**

|  | **Weighted kappa** | **PABAK** | **Gwet’s AC1** |
| --- | --- | --- | --- |
| **Hepatic vein** | 0.94 | 0.89 | 0.88 |
| **Portal vein** | 0.52 | 0.54 | 0.55 |
| **Intra-renal vein** | 0.92 | 0.84 | 0.86 |
| **VExUS** | 0.86 | 0.72 | 0.78 |

**Supplementary Table 3:** **Evolution of ultrasound parameters from enrolment to Day 3**

|  | **First assessment (Enrolment) (N=100)** | **Second assessment (day 3) (N = 80)** | **All assessments (N = 180)** | **P-value** |
| --- | --- | --- | --- | --- |
| **Hepatic vein Doppler** |  |  |  |  |
| Grade 0-1 | 46 (46%) | 38 (47.5%) | 84 (46.6%) | 1 |
| Grade 2-3 | 54 (54%) | 42 (52.5%) | 96 (53.3%) |  |
| **Portal vein Doppler** |  |  |  |  |
| Median PPF (IQR) | 34.2 (24.3-41.9) | 31.1 (23.4-39.1) | 33.3 (23.8-41) | 0.51 |
| Grade 0-1 | 38 (38%) | 36 (45%) | 74 (41.1%) | 0.58 |
| Grade 2-3 | 62 (62%) | 44 (55%) | 106 (58.8%) |  |
| **Renal venous Doppler** |  |  |  |  |
| Grade 0-1 | 37 (37%) | 24 (30%) | 61 (33.8%) | 0.28 |
| Grade 2-3 | 63 (63%) | 56 (70%) | 119 (66.1%) |  |
| **VExUS grading** |  |  |  |  |
| Grade 0-1 | 80 (80%) | 59 (73.7%) | 139 (77.2%) | 0.25 |
| Grade 2 | 6 (6%) | 11 (13.7%) | 17 (9.4%) |  |
| Grade 3 | 14 (14%) | 10 (12.5%) | 24 (13.3%) |  |
| **Modified VExUS grading** |  |  |  |  |
| Grade 0-1 | 65 (65%) | 47 (58.7%) | 112 (62.2%) | 0.42 |
| Grade 2 | 12 (12%) | 17 (21.2%) | 29 (16.1%) |  |
| Grade 3 | 23 (23%) | 16 (20%) | 39 (21.6%) |  |
| **IVC** |  |  |  |  |
| Maximal diameter (cm) | 1.8 (1.56-2.21) | 1.82 (1.33-2.12) | 1.81 (1.42-2.15) | 0.036 |
| Distensibility (Median IQR) | 13.9 (6.5-25.6) | 13.8 (5.8-23.3) | 13.8 (6.3-24.6) | 0.88 |
| **Median Lung US score (IQR)** | 4 (1-9) | 3 (1-9) | 3.5 (1-9) | 0.54 |

**Abbreviations:** *IQR* interquartile range, *IVC* inferior vena cava, *PF* pulsatility fraction, *US* ultrasound, *VExUS* venous excess ultrasound score

**Supplementary Table 4: Patient characteristics by mVExUS grading at enrolment**

| **Variables** | **Overall (N=100)** | **mVExUS**  **Grade 0-1 (n=65)** | **mVExUS**  **Grade 2-3 (n=35)** | **p-value** |
| --- | --- | --- | --- | --- |
| **Demographics** |  |  |  |  |
| Age (years) | 66 (51-77) | 66 (49-76) | 72 (56-80) | 0.06 |
| Male sex | 58 (58%) | 37 (56.9%) | 21 (60%) | 0.76 |
| Medical ICU admission | 82 (82%) | 55 (84.6%) | 27 (77.1%) | 0.78 |
| **Comorbidities** |  |  |  |  |
| Chronic kidney disease | 48 (48%) | 29 (44.6%) | 19 (54.2%) | 0.35 |
| eGFR baseline (mL/min/1.73m^2^) | 19.44 (13.8-32.7) | 19.4 (13.4-35.6) | 19.4 (14.4-27.1) | 0.94 |
| Hypertension | 55 (55) | 33 (50.7%) | 22 (62.8%) | 0.24 |
| Diabetes mellitus | 31 (31%) | 21 (32.3%) | 10 (28.5%) | 0.7 |
| Dyslipidemia | 53 (53%) | 34 (52.3%) | 19 (54.2%) | 0.85 |
| Autoimmune disease | 13 (13%) | 10 (15.3%) | 3 (8.5%) | 0.33 |
| Chronic lung disease | 8 (8%) | 4 (6.15%) | 4 (11.4%) | 0.35 |
| Chronic liver disease | 4 (4%) | 4 (6.15%) | 0 | 0.13 |
| Peripheral artery disease | 4 (4%) | 2 (3%) | 2 (5.7%) | 0.52 |
| Human immunodeficiency virus | 2 (2%) | 1 (1.5%) | 1 (2.8%) | 0.65 |
| Ischemic heart disease | 21 (21%) | 11 (16.9%) | 10 (28.5%) | 0.17 |
| Malignancy | 21 (21%) | 13 (20%) | 8 (22.8%) | 0.73 |
| Charlson comorbidity index | 4 (2-7) | 3 (2-7) | 6 (3-8) | 0.01 |
| Mechanical ventilation | 97 (97%) | 63 (96.9%) | 34 (97.1%) | 0.95 |
| Norepinephrine equivalent dose (mcg/kg/min) | 0.22 (0.08-0.52) | 0.27 (0.05-0.54) | 0.18 (0.1-0.49) | 0.91 |
| **Admission diagnosis** |  |  |  |  |
| Cardiovascular | 19 (19%) | 10 (15.3%) | 9 (25.7%) | 0.9 |
| Sepsis | 34 (34%) | 22 (33.8%) | 12 (34.2%) |  |
| Gastrointestinal/hepatic | 11 (11%) | 7 (10.7%) | 4 (11.4%) |  |
| Respiratory | 14 (14%) | 10 (15.3%) | 4 (11.4%) |  |
| Neurologic | 3 (3%) | 2 (3%) | 1 (2.8%) |  |
| Trauma | 4 (4%) | 3 (4.6%) | 1 (2.8%) |  |
| Other | 15 (15%) | 11 (16.9%) | 4 (11.4%) |  |
| Sepsis | 61 (61%) | 39 (60%) | 22 (62.8%) | 0.78 |
| APACHE II | 21.5 (15.5-26) | 20 (15-26) | 23 (18-26) | 0.11 |
| SOFA | 13 (10-15) | 12 (9-15) | 14 (10-16) | 0.13 |
| **Indications for RRT^a^** |  |  |  |  |
| Refractory acidosis | 44 (44%) | 32 (72.7%) | 12 (27.2%) | 0.15 |
| Refractory volume overload | 33 (33%) | 22 (66.7%) | 11 (33.3%) | 0.8 |
| Refractory hyperkalemia | 7 (7%) | 6 (85.7%) | 1 (14.3%) | 0.23 |
| Anuria, oliguria | 56 (56%) | 36 (64.3%) | 20 (35.7%) | 0.86 |
| Uremia | 17 (17%) | 9 (52.9%) | 8 (47.1%) | 0.25 |
| High BUN | 33 (33%) | 21 (63.6%) | 12 (36.4%) | 0.84 |
| **Echocardiographic parameters** |  |  |  |  |
| TAPSE (cm) (n=60) | 1.8 (1.5-2.1) | 2 (1.69-2.24) | 1.5 (1.01-1.65) | 0.0002 |
| LVEF (%) (n=73) | 56 (38-66) | 60 (44-66) | 44 (33-66) | 0.11 |
| VTI (cm) (n=58) | 15.05 (12-20.2) | 17.5 (12.8-22.2) | 12.7 (9.3-16.5) | 0.01 |
| LV fractional shortening (%) (n=75) | 30.2 (18.9-36.9) | 31.2 (21.8-37.9) | 23 (17.8-34.4) | 0.11 |
| MV E:A ratio (n=42) | 0.96 (0.71-1.5) | 0.83 (0.69-1.4) | 1.2 (0.8-1.7) | 0.25 |
| E/Septal E’ (n=62) | 13.9 (9.2-17.2) | 13.3 (8.8-16.7) | 14.5 (9.75-17.25) | 0.65 |
| E/Lateral E’ (n= 60) | 10.3 (7.05-13.8) | 9.8 (7-12.5) | 11.2 (8-16.5) | 0.15 |
| Tricuspid S’ vel (cm/sec) (n=62) | 11 (9-13.4) | 11.6 (10.4-14.3) | 8.85 (6.9-11.4) | 0.001 |
| RVSP (mmHg) (n=50) | 33.8 (28.3-42.4) | 32.2 (26.7-39.4) | 42 (30.7-52.2) | 0.02 |
| Central venous pressure (cmH_2_O) (n=86) | 14 (11-17) | 13 (10-16) | 16 (13-20) | 0.003 |
| Overhydration by BIA (L) | 4.65 (2.3-6.8) | 4 (3-6) | 5 (2-8) | 0.52 |
| Cumulative fluid balance at enrolment (%) | 3.49 (1.35-7.01) | 3.65 (1.21-7.65) | 2.15 (1.38-5.27) | 0.24 |
| **Laboratory parameters** |  |  |  |  |
| Hb (g/dL) | 8.5 (7.4-10.2) | 8.8 (7.5-11.1) | 8.4 (7.4-9.2) | 0.18 |
| WBC (/10^3^) | 14 (7.2-20.7) | 14.5 (7-20.5) | 13.9 (7.2-22.4) | 0.73 |
| Neutrophils (/10^3^) | 84.3 (76.1-90.5) | 83.8 (75-89.8%) | 86 (81-93.6) | 0.14 |
| Lymphocytes (/10^3^) | 7.05 (3.9-13.6) | 9 (4-14.7) | 4.5 (2.5-10.9) | 0.02 |
| Platelets (/10^3^/uL) | 119.5 (70-206.5) | 118 (80-226) | 121 (54-182) | 0.3 |
| BUN (mg/dL) | 57.5 (35.5-94.5) | 61 (38-94) | 48 (25-101) | 0.82 |
| Creatinine (mg/dL) | 2.99 (1.84-3.95) | 3.01 (1.84-3.97) | 2.97 (1.9-3.93) | 0.91 |
| Sodium (mEq/L) | 138 (135-143) | 138 (135-143) | 139 (134-144) | 0.78 |
| Potassium (mEq/L) | 4.15 (3.75-4.85) | 4 (3.8-4.9) | 4.2 (3.6-4.7) | 0.75 |
| Chloride (mEq/L) | 101 (97-106) | 101 (97-106) | 101 (97-105) | 0.67 |
| Carbon dioxide (mEq/L) | 16 (11.5-20) | 16 (11-20) | 17 (12-21) | 0.41 |
| Lactate (mg/dL) | 5.15 (2-11) | 5.4 (2.2-11.2) | 3.8 (1.8-10.3) | 0.91 |
| Urinary NGAL (ng/mL) (n=62) | 1809 (267-4327.6) | 1011 (231-4327) | 1939 (905-4236) | 0.33 |
| NT-proBNP (pg/mL) (n=95) | 14661 (3905-35000) | 10346 (2104-29404) | 21056 (9822-35000) | 0.01 |

**Abbreviations:** *AKI* acute kidney injury, *APACHE II* Acute Physiology and Chronic Health Evaluation, *BIA* Bioelectrical Impedance Analysis, *BUN* blood urea nitrogen, *Cr* creatinine, *eGFR* estimated glomerular filtration rate, *Hb* Hemoglobin, *IQR* interquartile range, *IVC* inferior vena cava, *LVEF* left ventricular ejection fraction, *NGAL* neutrophil gelatinase-associated lipocalin, *NT-proBNP* N-terminal pro B-type natriuretic peptide, *SD* standard deviation, *SOFA* sequential organ failure assessment, *TAPSE* tricuspid annulus plane systolic exertion, *VTI* velocity time integral, *WBC* white blood cell

^a^ Indications for RRT: refractory acidosis (pH < 7.2, HCO_3_^-^ < 15 mEq/L), refractory volume overload (unresponsive to diuretics), anuria, oliguria (urine output less than 0.5 ml/kg/h for 6-12 hrs), uremic symptom and sign (mental status change, pericardial friction rub, intractable nausea/vomiting, myoclonus, seizure not attributable to another etiology), high BUN >112 mg/dL

**Supplementary Table 5:** **Patient characteristics and parameters at enrolment in relationship with 90-day mortality**

| **Variables** | **Overall (N=100)** | **Survivors (N= 34)** | **Non-Survivors (N= 66)** | **p-value** |
| --- | --- | --- | --- | --- |
| Age (Median IQR) | 66 (51-77) | 55.5 (49-70) | 71.5 (56-79) | 0.01 |
| Male sex | 58 | 18 (31%) | 40 (68.9%) | 0.46 |
| Body weight (kg) | 62.8 (50.5-70) | 64.5 (54-75.6) | 60.05 (49.4-68) | 0.09 |
| Medical ICU admission | 82 | 29 (35.3%) | 53 (64.6%) | 0.84 |
| Cardiac surgery | 6 | 1 (2.9%) | 5 (7.5%) | 0.41 |
| Time to CRRT initiation from ICU admission (days) | 1 (0-3) | 1 (0-2) | 1 (0-3) | 0.45 |
| **Comorbidities** |  |  |  |  |
| Chronic kidney disease | 48 | 16 (33.3%) | 32 (66.6%) | 0.89 |
| eGFR baseline | 49.52 (30.5-76.83) | 49.22 (30.5-80.1) | 49.52 (30.6-76.29) | 0.69 |
| Hypertension | 55 | 18 (32.7%) | 37 (62.7%) | 0.76 |
| Diabetes mellitus | 31 | 13 (41.9%) | 18 (58%) | 0.26 |
| Dyslipidemia | 53 | 17 (32%) | 36 (67.9%) | 0.66 |
| Autoimmune disease | 13 | 6 (46.1%) | 7 (53.8%) | 0.32 |
| Chronic lung disease | 8 | 1 (12.5%) | 7 (87.5%) | 0.18 |
| Chronic liver disease | 4 | 1 (25%) | 3 (75%) | 0.69 |
| Peripheral artery disease | 4 | 1 (25%) | 3 (75%) | 0.69 |
| Human immunodeficiency virus | 2 | 0 | 2 (100%) | 0.3 |
| Ischemic heart disease | 21 | 4 (19%) | 17 (80.9%) | 0.1 |
| Malignancy | 21 | 6 (28.5%) | 15 (71.4%) | 0.55 |
| Charlson comorbidity index | 4 (2-7) | 3 (2-5) | 5 (3-8) | 0.06 |
| Mechanical ventilation | 97 (97%) | 32 (32.9%) | 65 (67%) | 0.22 |
| Norepinephrine equivalent dose (mcg/kg/min) | 0.22 (0.08-0.52) | 0.11 (0-0.52) | 0.28 (0.11-0.57) | 0.01 |
| **Admission diagnostic criteria** | | | | |
| Cardiovascular | 19 | 6 (31.5%) | 13 (18.4%) | 0.49 |
| Sepsis | 34 | 11 (32.3%) | 23 (67.6%) |  |
| Gastrointestinal/hepatic | 11 | 3 (27.2%) | 8 (72.7%) |  |
| Respiratory | 14 | 3 (21.4%) | 11 (78.5%) |  |
| Neurologic | 3 | 1 (33.3%) | 2 (66.6%) |  |
| Trauma | 4 | 3 (75%) | 1 (25%) |  |
| Other | 15 | 7 (46.6%) | 8 (53.3%) |  |
| APACHE II (Median IQR) | 21.5 (15.5-26) | 19.5 (15-25) | 22 (16-26) | 0.17 |
| SOFA (Median IQR) | 13 (10-15) | 11.5 (8-14) | 14 (11-15) | 0.04 |
| **Causes of AKI** | | |  |  |
| Haemodynamic | 33 (33%) | 9 (27.2%) | 24 (72.7%) | 0.15 |
| Nephrotoxic | 7 (7%) | 4 (57.1%) | 3 (42.8%) |  |
| Sepsis | 34 (34%) | 10 (29.4%) | 24 (70.5%) |  |
| Cardiorenal | 24 (24%) | 9 (37.5%) | 15 (62.5%) |  |
| Other | 2 (2%) | 2 (100%) | 0 |  |
| **Indication for RRT^a^** | | | | |
| Refractory acidosis | 44 (44%) | 21 (47.8% | 23 (52.2%) | 0.75 |
| Refractory volume overload | 33 (33%) | 17 (51.6%) | 16 (48.4%) | 0.43 |
| Refractory hyperkalemia | 7 (7%) | 3 (42.8%) | 4 (57.1%) | 0.86 |
| Anuria, oliguria | 56 (56%) | 25 (44.6%) | 31 (55.3%) | 0.75 |
| Uremia | 17 (17%) | 9 (52.9%) | 8 (47%) | 0.52 |
| High BUN | 33 (33%) | 17 (51.5%) | 16 (48.4%) | 0.43 |
| **Echocardiographic parameters** |  |  |  |  |
| TAPSE (cm) (n=60) | 1.8 (1.5-2.1) | 2.03 (1.6-2.3) | 1.75 (1.37-2.1) | 0.1 |
| LVEF (%) (n=73) | 56 (38-66) | 62 (43-67) | 47.5 (37-64.5) | 0.35 |
| VTI (cm) (n=58) | 15.05 (12-20.2) | 17.7 (13.4-20.8) | 13.4 (11.3-18.9) | 0.16 |
| **Laboratory parameters at enrollment** |  |  |  |  |
| Hb (g/dL) | 8.5 (7.4-10.2) | 8.45 (7.5-10.6) | 8.55 (7.3-9.6) | 0.32 |
| WBC (/10^3^) | 14 (7.2-20.7) | 14.5 (9.39-17.8) | 13.4 (5.9-22.8) | 0.7 |
| Neutrophils (/10^3^) | 84.3 (76.1-90.5) | 84.8 (77-93.6) | 84 (76-89.4) | 0.29 |
| Lymphocytes (/10^3^) | 7.05 (3.9-13.6) | 7.35 (3.9-13.6) | 7.05 (3.9-13.6) | 0.75 |
| Platelets (/10^3^/uL) | 119.5 (70-206.5) | 129.5 (78-244) | 116.5 (67-204) | 0.4 |
| BUN (mg/dL) | 57.5 (35.5-94.5) | 63 (40-94) | 56.5 (29-95) | 0.64 |
| Creatinine (mg/dL) | 2.99 (1.84-3.95) | 3.06 (2.06-4.19) | 2.99 (1.9-3.84) | 0.26 |
| Sodium (mEq/L) | 138 (135-143) | 136.5 (134-141) | 139 (135-144) | 0.08 |
| Potassium (mEq/L) | 4.15 (3.75-4.85) | 4.05 (3.7-5) | 4.2 (3.8-4.8) | 0.97 |
| Chloride (mEq/L) | 101 (97-106) | 100 (97-106) | 101 (97-106) | 0.89 |
| Carbon dioxide (mEq/L) | 16 (11.5-20) | 16 (10-21) | 17 (12-20) | 0.45 |
| Lactate (mg/dL) | 5.15 (2-11) | 3.7 (1-10) | 6 (2.4-11.2) | 0.07 |
| Urinary NGAL (ng/mL) (n=62) | 1809 (267-4327.6) | 2449 (697-5662) | 1448.5 (211.7-3804.4) | 0.14 |
| **Fluid status parameters** |  |  |  |  |
| NT-proBNP (pg/mL) | 14661 (3905-35000) | 8340 (1952-30879) | 15868 (7134-35000) | 0.25 |
| Central venous pressure (cmH_2_O) | 14 (11-17) | 14 (11.5-16) | 13.5 (11-18) | 0.07 |
| Overhydration by BIVA (L) | 4.65 (2.3-6.8) | 4 (1-6) | 5 (3-6) | 0.22 |
| Cumulative fluid balance at enrollment (mL) | 2157 (692.5-4107.5)) | 1862.5 (484-3661) | 2203.5 (992-4180) | 0.41 |
| Cumulative fluid balance at day 3 (mL) | 4547 (714-7608) | 3481 (-800-6274) | 5271 (961-9356) | 0.06 |
| Lung ultrasound scores | 4 (1-9) | 3.5 (0-10) | 4 (1-9) | 0.6 |
| **VEXUS Grading** |  |  |  |  |
| Grade 0-1 | 80 (80%) | 30 (37.5%) | 50 (62.5%) | 0.13 |
| Grade 2-3 | 20 (20%) | 4 (20%) | 16 (80%) |  |
| **mVExUS Grading** |  |  |  |  |
| Grade 0-1 | 65 (65%) | 26 (40%) | 39 (60%) | 0.08 |
| Grade 2-3 | 35 (35%) | 8 (22.8%) | 27 (77.1%) |  |

**Abbreviations:** *AKI* acute kidney injury, *APACHE II* Acute Physiology and Chronic Health Evaluation, *BIVA* Bioelectrical Impedance Vector Analysis, *BUN* blood urea nitrogen, *Cr* creatinine, *eGFR* estimated glomerular filtration rate, *Hb* Hemoglobin, *IQR* interquartile range, *IVC* inferior vena cava, *LVEF* left ventricular ejection fraction, *NGAL* neutrophil gelatinase-associated lipocalin, *NT-proBNP* N-terminal pro B-type natriuretic peptide, *SD* standard deviation, *SOFA* sequential organ failure assessment, *TAPSE* tricuspid annulus plane systolic exertion, *VTI* velocity time integral, *WBC* white blood cell

* Fisher’s exact test

^a^ Indications for RRT: refractory acidosis (pH < 7.2, HCO_3_^-^ < 15 mEq/L), refractory volume overload (unresponsive to diuretics), anuria, oliguria (urine output less than 0.5 ml/kg/h for 6-12 hrs), uremic symptom and sign (mental status change, pericardial friction rub, intractable nausea/vomiting, myoclonus, seizure not attributable to another etiology), high BUN >112 mg/dL

**Supplementary Table 6:** **The association between ultrasound markers as time-dependent variables and mortality through 90 days**

|  | **Univariable HR (95% CI) p-value** | **Multivariable* Adjusted HR (95% CI) p-value** |
| --- | --- | --- |
| Hepatic vein Doppler |  |  |
| Grade 0-1 | Ref | Ref |
| Grade 2-3 | 1.85 (1.1-3.12) 0.01 | 1.78 (1.05-3.01) 0.01 |
| Portal vein Doppler |  |  |
| Grade 0-1 | Ref | Ref |
| Grade 2-3 | 1.09 (0.66-1.8) 0.73 | 1.12 (0.68-1.86) 0.64 |
| Per 10% increase in pulsatility fraction | 1.008 (0.99-1.02) 0.18 | 1.01 (0.99-1.02) 0.13 |
| Intra-renal vein Doppler |  |  |
| Grade 0-1 | Ref | Ref |
| Grade 2-3 | 1.2 (0.7-2.06) 0.49 | 1.13 (0.65-1.95) 0.65 |
| mVExUS grading |  |  |
| Grade 0-1 | Ref | Ref |
| Grade 2-3 | 1.47 (1.01-2.13) 0.04 | 1.38 (0.94-2.01) 0.09 |
| VExUS |  |  |
| Grade 0-1 | Ref | Ref |
| Grade 2-3 | 1.48 (0.97-2.25) 0.06 | 1.41 (0.93-2.15) 0.1 |
| **LUS scores** | 1.05 (1.008-1.09) 0.02 | 1.04 (1.001-1.08) 0.04 |

* adjusted for SOFA score

**Abbreviation:** *CI* confidence interval, *HR* hazard ratio, *mVExUS* modified venous excess ultrasound score, *VExUS* venous excess ultrasound score

**Supplementary Table 7: Comparison of the C-statistics, NRI, and IDI of VExUS versus mVExUS in predicting 90-day mortality**

|  | **C-Statistics** | **P value** | **NRI (95% CI)** | **P value** | **IDI (95% CI)** | **P value** |
| --- | --- | --- | --- | --- | --- | --- |
| **VExUS** | 0.6 | 0.22 | 0.2 (-0.12-0.53) | 0.22 | 0.02 (-0.00007-0.59) | 0.049 |
| **mVExUS** | 0.65 |  |  |  |  |  |

**Abbreviations:** CI, confidence interval; IDI, integrated discriminatory index; mVExUS, modified venous excess ultrasound; NRI, net reclassification index, VExUS, venous excess ultrasound

**Supplementary Table 8: Association between VExUS and mVExUS grading at baseline and secondary outcomes**

| **Outcomes** | **VExUS** | | **mVExUS** | |
| --- | --- | --- | --- | --- |
|  | **HR/RRR/OR (95% CI)*** | **P value** | **HR/RRR/OR (95% CI)*** | **P value** |
| Death at 28 days | **HR**  Grade 0-1 : Ref  Grade 2-3: 1.64 (0.89-3.03) | 0.1 | **HR**  Grade 0-1: Ref  Grade 2-3: 1.27 (1.005-1.62) | 0.045 |
| Death at 90 days | **HR**  Grade 0-1: Ref  Grade 2-3: 1.73 (0.98-3.06) | 0.15 | **HR**  Grade 0-1: Ref  Grade 2-3: 1.78 (1.08-2.91) | 0.024 |
| Dialysis dependence at 28 days | **RRR**  Grade 0-1: Ref  Grade 2-3: 0.91 (0.55-1.49) | 0.68 | **RRR**  Grade 0-1: Ref  Grade 2-3: 0.79 (0.29-2.12) | 0.64 |
| Dialysis dependence at 90 days | **RRR**  Grade 0-1: Ref  Grade 2-3: 0.97 (0.44-2.16) | 0.93 | **RRR**  Grade 0-1: Ref  Grade 2-3: 0.08 (-1.05-1.22) | 0.88 |
| ICU-free days | Grade 0-1: 0 (0; 18)  Grade 2-3: 0 | 0.08 | Grade 0-1: 0 (0; 18)  Grade 2-3: 0 (0; 9) | 0.08 |
| Vasopressor-free days | Grade 0-1: 18.5 (0; 24)  Grade 2-3: 0 (0; 20) | 0.06 | Grade 0-1: 19 (0; 25)  Grade 2-3: 0 (0; 22) | 0.04 |
| Ventilator-free days | Grade 0-1: 0 (0; 19.5)  Grade 2-3: 0 (0; 18.5) | 0.41 | Grade 0-1: 0 (0; 23)  Grade 2-3: 0 (0; 18) | 0.09 |
| RRT-free days | Grade 0-1: 0 (0; 12)  Grade 2-3: 0 (0; 17) | 0.94 | Grade 0-1: 0 (0; 15.5)  Grade 2-3: 0 (0; 12) | 0.4 |

*Death was assessed using Cox regression analysis. MAKE and in-hospital mortality were assessed using univariable binary logistic regression. Persistent kidney dysfunction and RRT dependence were assessed using multinomial logistic regression to account for the competing risk of death.

**Abbreviations:** *CI* confidence interval, *HR* hazard ratio, *ICU* intensive care unit, *MAKE* major adverse kidney event, *mVExUS* modified venous excess ultrasound score, *OR* Odds ratio, *RRR* relative risk reduction, *RRT* renal replacement therapy, *VExUS* venous excess ultrasound score

**Supplementary Table 9:** **Association between LUS scores on Day 1 and 3 and outcomes**

| **Outcomes** | **Day 1** | **P-value** | **Day 3** | **P value** |
| --- | --- | --- | --- | --- |
|  | **HR/RRR/OR (95% CI)*** |  | **HR/RRR/OR (95% CI)*** |  |
| 28-day mortality | 1.003 (0.95-1.05) | 0.88 | 1.08 (1.031-1.13) | 0.001 |
| 90-day mortality | 1.007 (0.96-1.05) | 0.73 | 1.08 (1.04-1.13) | <0.001 |
| RRT dependence at 28 days | 1.07 (0.99-1.17) | 0.07 | 0.95 (0.86-1.05) | 0.38 |
| RRT dependence at 90 days | 1.12 (1.01-1.24) | 0.02 | 0.96 (0.84-1.09) | 0.53 |
| ICU-free days through day 28 (IQR) | ≥4: 0 (0; 16)  <4: 0 (0; 17) | 0.66 | ≥3: 11 (0; 20)  <3: 0 (0; 15) | 0.01 |
| Vasopressor-free days (IQR) | ≥4: 14 (0; 24)  <4: 0 (0; 24) | 0.63 | ≥3: 0 (0; 23)  <3: 0 (0) | 0.01 |
| Ventilator-free days (IQR) | ≥4: 0 (0; 23)  <4: 0 (0; 18) | 0.08 | ≥3: 17 (0; 23)  <3: 0 (0; 19) | 0.008 |
| RRT-free days (IQR) | ≥4: 0 (0; 19)  <4: 0 (0; 6) | 0.32 | ≥3: 0 (0; 15)  <3: 0 (0; 0) | 0.16 |

**Abbreviations:** *CI* confidence interval, *HR* hazard ratio, *ICU* intensive care unit, *IQR* interquartile range, *MAKE* major adverse kidney event, *RR* relative risk, *RRT* renal replacement therapy

**Supplementary Table 10: Sensitivity analysis: univariable and multivariable Cox regression analysis of VExUS and mVExUS scores at baseline as ordinal variables and 90-day mortality**

| **Variables** | **HR (95% CI)** | **P value** | **aHR (95% CI)*** | **P value** |
| --- | --- | --- | --- | --- |
| **VExUS** |  |  |  |  |
| **Grade 0** | Reference |  | Reference |  |
| **Grade 1** | 0.59 (0.27-1.26) | 0.17 | 0.6 (0.28-1.3) | 0.2 |
| **Grade 2** | 2.72 (1.04-7.1) | 0.04 | 2.47 (0.94-6.52) | 0.06 |
| **Grade 3** | 1.31 (0.67-2.56) | 0.41 | 1.3 (0.67-2.54) | 0.43 |
| **mVExUS** |  |  |  |  |
| **Grade 0** | Reference |  | Reference |  |
| **Grade 1** | 0.99 (0.52-1.89) | 0.98 | 0.98 (0.51-1.88) | 0.97 |
| **Grade 2** | 1.53 (0.65-3.6) | 0.32 | 1.37 (0.58-3.27) | 0.46 |
| **Grade 3** | 1.88 (0.96-3.67) | 0.06 | 1.79 (0.92-3.5) | 0.08 |

*****adjusted with baseline Sequential Organ Failure Assessment

**Abbreviations:** CI, confidence interval; HR, hazard ratio, mVExUS, modified venous excess ultrasound; VExUS, venous excess ultrasound

**Supplementary Table 11: Sensitivity analysis: Cox proportional hazard analysis of associations between variables at enrolment and 90-day mortality in patients without chronic liver disease (n=96)**

| **Variables** | **HR (95% CI)** | **P value** | **aHR (95% CI)*** | **P value** | **aHR (95% CI)*** | **P value** |
| --- | --- | --- | --- | --- | --- | --- |
| **Age** | 1.01 (0.99-1.02) | 0.09 |  |  |  |  |
| **Male sex** | 0.89 (0.53-1.49) | 0.66 |  |  |  |  |
| **Norepinephrine equivalent dose (mcg/kg/min)** | 1.46 (0.97-2.2) | 0.06 |  |  |  |  |
| **SOFA score** | 1.05 (0.98-1.11) | 0.11 | 1.03 (0.97-1.1) | 0.23 | 1.05 (0.99-1.12) | 0.11 |
| **Lactate (mmol/L)** | 1.04 (1.001-1.08) | 0.04 |  |  |  |  |
| **LUS score** | 1.01 (0.96-1.05) | 0.63 |  |  |  |  |
| **CVP (cmH2O)** | 1.02 (0.97-1.07) | 0.33 |  |  |  |  |
| **OH by BIA (per 1 L)** | 1.05 (0.97-1.14) | 0.16 |  |  |  |  |
| **NT-proBNP** | 1.000006 (0.99-1.000025) | 0.56 |  |  |  |  |
| **CFB (per 1 L** | 1.009 (0.92-1.1) | 0.84 |  |  |  |  |
| **VExUS** |  |  |  |  |  |  |
| **Grade 0-1** | Ref |  |  |  | Ref |  |
| **Grade 2-3** | 1.8 (1.01-3.21) | 0.04 |  |  | 1.67 (0.95-2.95) | 0.08 |
| **mVExUS** |  |  |  |  |  |  |
| **Grade 0-1** | Ref |  | Ref |  |  |  |
| **Grade 2-3** | 1.87 (1.13-3.1) | 0.01 | 1.77 (1.06-2.95) | 0.02 |  |  |

**Abbreviations:** CFB, cumulative fluid balance; CI, confidence interval; CVP, central venous pressure; HR, hazard ratio; mVExUS, modified venous excess ultrasound; NT-proBNP N-terminal pro B-type natriuretic peptide; OH, overhydration; SOFA, Sequential Organ Failure Assessment; VExUS, venous excess ultrasound

**Supplementary Table 12:** **Sensitivity analysis: association between VExUS and mVExUS grading on Day 3 and secondary outcomes**

| **Outcomes** | **VExUS** |  | **mVExUS** |  |
| --- | --- | --- | --- | --- |
|  | **HR/RRR/OR (95% CI)*** | **P value** | **HR/RRR/OR (95% CI)*** | **P value** |
| Death at 28 days | **HR**  Grade 0-1: Ref  Grade 2-3: 1.43 (0.7-2.91) | 0.42 | **HR**  Grade 0-1: Ref  Grade 2-3: 1.41 (0.73-2.73) | 0.29 |
| Death at 90 days | **HR**  Grade 0-1: Ref  Grade 2-3: 1.31 (0.7-2.43) | 0.6 | **HR**  Grade 0-1: Ref  Grade 2-3: 1.21 (0.69-2.14) | 0.49 |
| Dialysis dependence at 28 days | **RRR**  Grade 0-1: Ref  Grade 2-3: 0.26 (0.05-1.24) | 0.09 | **RRR**  Grade 0-1: Ref  Grade 2-3: 0.62 (0.37-1.04) | 0.07 |
| Dialysis dependence at 90 days | **RRR**  Grade 0-1: Ref  Grade 2-3: 0.27 (0.03-2.33) | 0.23 | **RRR**  Grade 0-1: Ref  Grade 2-3: 0.82 (0.44-1.53) | 0.54 |
| ICU-free days | Grade 0-1: 0 (0; 18)  Grade 2-3: 0 (0; 20) | 0.62 | Grade 0-1: 0 (0;18)  Grade 2-3: 0 (0;20) | 0.85 |
| Vasopressor-free days | Grade 0-1: 21 (0; 25)  Grade 2-3: 0 (0; 23) | 0.11 | Grade 0-1:21 (0;26)  Grade 2-3: 13 (0;23) | 0.12 |
| Ventilator-free days | Grade 0-1: 0 (0; 20)  Grade 2-3: 0 (0;23) | 0.87 | Grade 0-1:0 (0;20)  Grade 2-3: 0 (0;23) | 0.97 |
| RRT-free days | Grade 0-1: 0 (0; 16)  Grade 2-3: 0 (0; 19) | 0.87 | Grade 0-1:0 (0;15)  Grade 2-3: 0 (0;20) | 0.24 |

*Death was assessed using Cox regression analysis. MAKE and in-hospital mortality were assessed using univariable binary logistic regression. Persistent kidney dysfunction and RRT dependence were assessed using multinomial logistic regression to account for the competing risk of death.

**Abbreviations:** *CI* confidence interval, *HR* hazard ratio, *ICU* intensive care unit, *MAKE* major adverse kidney event, *OR* Odds ratio, *RRR* relative risk reduction, *RRT* renal replacement therapy, *VExUS* venous excess ultrasound score

**Supplementary Table 13a: Spearman’s Correlation between parameters to assess fluid status**

| -0.0127  P=0.85 | -0.0056  P=0.88 | 0.0402  P=0.8 | 0.3965  P=0.64 | 0.1601  P=0.96 | 0.1061  P=0.29 | -0.2145*  P=0.005 | 0.1095  P=0.85 | 0.5044*  P<0.001 | 0.1088  P=0.84 | -0.1834  P=0.34 | 1 | **CFB** |  |
| --- | --- | --- | --- | --- | --- | --- | --- | --- | --- | --- | --- | --- | --- |
| 0.0510  P=0.61 | 0.1216  P=0.22 | 0.1738  P=0.08 | 0.2189*  P=0.04 | 0.2897*  P=0.003 | 0.4029*  P<0.001 | -0.0181  P=0.76 | 0.0880  P=0.31 | 0.2417*  P=0.01 | -0.1250  P=0.34 | 1 | -0.1834  P=0.34 | **LUS** |  |
| 0.1058  P=0.88 | -0.0017  P=0.7 | -0.3611*  P=0.005 | -0.051  P=0.38 | -0.3282*  P=0.003 | -0.383*  P=0.001 | -0.167  P=0.32 | -0.429*  P=0.001 | 0.2372  P=0.69 | 1 | -0.1250  P=0.34 | 0.1088  P=0.84 | **TAPSE** |  |
| 0.0958  P=0.11 | 0.0888  P=0.66 | -0.0838  P=0.77 | 0.2691  P=0.09 | 0.1076  P=0.34 | -0.1173  P=0.99 | 0.0481  P=0.76 | -0.2096  P=0.63 | 1 | 0.2372  P=0.69 | 0.2417*  P=0.01 | 0.5044*  P<0.001 | **BIA** |  |
| 0.0273  P=0.76 | 0.0543  P=0.97 | 0.1174  P=0.22 | 0.0248  P=0.57 | 0.1839*  P=0.01 | 0.3818*  P<0.001 | 0.236  P=0.35 | 1 | -0.2096  P=0.63 | -0.429*  P=0.001 | 0.0880  P=0.31 | 0.1095  P=0.85 | **NT-proBNP** |  |
| 0.1877*  P=0.03 | 0.2169*  P=0.01 | 0.1519*  P=0.009 | 0.29*  P<0.001 | 0.3138*  P=0.003 | 0.1545*  P=0.002 | 1 | 0.236  P=0.35 | 0.0481  P=0.76 | -0.167  P=0.32 | -0.0181  P=0.76 | -0.2145*  P=0.005 | **PPF** |  |
| -0.119*  P=0.008 | 0.0184*  P=0.003 | 0.352*  P<0.001 | 0.3125*  P=0.001 | 0.48*  P<0.001 | 1 | 0.1545*  P=0.002 | 0.3818*  P<0.001 | -0.1173  P=0.99 | -0.383*  P=0.001 | 0.4029*  P<0.001 | 0.1061  P=0.29 | **HVF** |  |
| 0.1918*  P=0.01 | 0.2816*  P=0.004 | 0.4877*  P<0.001 | 0.5704*  P<0.001 | 1 | 0.48*  P<0.001 | 0.3138*  P=0.003 | 0.1839*  P=0.01 | 0.1076  P=0.34 | -0.3282*  P=0.003 | 0.2897*  P=0.003 | 0.1601  P=0.96 | **IRVF** |  |
| 0.1268*  P=0.003 | 0.1614*  P=0.002 | 0.1385*  P=0.007 | 1 | 0.5704*  P<0.001 | 0.3125*  P=0.001 | 0.29*  P<0.001 | 0.0248  P=0.57 | 0.2691  P=0.09 | -0.051  P=0.38 | 0.2189*  P=0.04 | 0.3965  P=0.64 | **CVP** |  |
| 0.4006*  P<0.001 | 0.499*  P<0.001 | 1 | 0.1385*  P=0.007 | 0.4877*  P<0.001 | 0.352*  P<0.001 | 0.1519*  P=0.009 | 0.1174  P=0.22 | -0.0838  P=0.77 | -0.3611*  P=0.005 | 0.1738  P=0.08 | 0.0402  P=0.8 | **mVExUS** |  |
| 0.9664*  P<0.001 | 1 | 0.499*  P<0.001 | 0.1614*  P=0.002 | 0.2816*  P=0.004 | 0.0184*  P=0.003 | 0.2169*  P=0.01 | 0.0543  P=0.97 | 0.0888  P=0.66 | -0.0017  P=0.7 | 0.1216  P=0.22 | -0.0056  P=0.88 | **VExUS** |  |
| 1 | 0.9664*  P<0.001 | 0.4006*  P<0.001 | 0.1268*  P=0.003 | 0.1918*  P=0.01 | -0.119*  P=0.008 | 0.1877*  P=0.03 | 0.0273  P=0.76 | 0.0958  P=0.11 | 0.1058  P=0.88 | 0.0510  P=0.61 | -0.0127  P=0.85 | **IVC** |  |
| **IVC** | **VExUS** | **mVExUS** | **CVP** | **IRVF** | **HVF** | **PPF** | **NT-proBNP** | **BIA** | **TAPSE** | **LUS** | **CFB** |  |  |
|  |  |  |  |  |  |  |  |  |  |  |  |  |  |

**0.00 to 0.19:** Very weak**; 0.20 to 0.39:** Weak**; 0.40 to 0.59:** Moderate**; 0.60 to 0.79:** Strong**; 0.80 to 1.0:** Very strong

**Abbreviations:** *BIA* bioelectrical impedance analysis, *CFB* cumulative fluid balance, *CVP* central venous pressure, *HVF* hepatic vein flow pattern, *IRVF* intra-renal vein flow pattern, *IVC* inferior vena cava, *LUS* lung ultrasound, *mVExUS* modified venous excess ultrasound score , *NT-proBNP* N-terminal pro B-type natriuretic peptide, *PPF* portal vein pulsatility fraction, *TAPSE* tricuspid annulus plane systolic exertion, *VExUS* venous excess ultrasound score

**Supplementary Table 13b: Number of paired observations in Spearman’s Correlation between parameters to assess fluid status**

| 100 | 100 | 100 | 86 | 100 | 100 | 100 | 98 | 94 | 60 | 100 | - | **CFB** |
| --- | --- | --- | --- | --- | --- | --- | --- | --- | --- | --- | --- | --- |
| 100 | 100 | 100 | 86 | 100 | 100 | 100 | 98 | 94 | 60 | - | 100 | **LUS** |
| 60 | 60 | 60 | 55 | 60 | 60 | 60 | 59 | 55 | - | 60 | 60 | **TAPSE** |
| 94 | 94 | 94 | 80 | 94 | 94 | 94 | 93 | - | 55 | 94 | 94 | **BIA** |
| 98 | 98 | 98 | 84 | 98 | 98 | 98 | - | 93 | 59 | 98 | 98 | **NT-proBNP** |
| 100 | 100 | 100 | 86 | 100 | 100 | - | 98 | 94 | 60 | 100 | 100 | **PPF** |
| 100 | 100 | 100 | 86 | 100 | - | 100 | 98 | 94 | 60 | 100 | 100 | **HVF** |
| 100 | 100 | 100 | 86 | - | 100 | 100 | 98 | 94 | 60 | 100 | 100 | **IRVF** |
| 86 | 86 | 86 | - | 86 | 86 | 86 | 84 | 80 | 55 | 86 | 86 | **CVP** |
| 100 | 100 | - | 86 | 100 | 100 | 100 | 98 | 94 | 60 | 100 | 100 | **mVExUS** |
| 100 | - | 100 | 86 | 100 | 100 | 100 | 98 | 94 | 60 | 100 | 100 | **VExUS** |
| - | 100 | 100 | 86 | 100 | 100 | 100 | 98 | 94 | 60 | 100 | 100 | **IVC** |
| **IVC** | **VExUS** | **mVExUS** | **CVP** | **IRVF** | **HVF** | **PPF** | **NT-proBNP** | **BIA** | **TAPSE** | **LUS** | **CFB** |  |

**References**

1.	Romano, M., E. Viana, J.D. Martins, and R. Corga Da Silva, *Evaluation Of Congestion Levels in Septic Patients Admitted to Critical Care Units with a Combined Venous Excess-Lung Ultrasound Score (VExLUS) - a Research Protocol.* Pocus j, 2023. **8**(1): p. 93-98.
